# On-Slide Preparation of *Caenorhabditis elegans* toward Quantitative, High-Resolution LA-ICP-TOF Mass Spectrometry Imaging

**DOI:** 10.1021/acs.analchem.6c04271

**Published:** 2026-09-17

**Authors:** Aidan J. Reynolds, Aaron Sue, Keith MacRenaris, Thomas V. O’Halloran, Tian Autumn Qiu

**Affiliations:** 1 Department of Chemistry, 3078 Michigan State University, East Lansing, Michigan 48824, United States; 2 Department of Microbiology, Genetics and Immunology, Michigan State University, East Lansing, Michigan 48824, United States; 3 Elemental Health Institute, Michigan State University, East Lansing, Michigan 48824, United States

## Abstract

Metal homeostasis is essential for biological functions and has been implicated in many diseases. Laser ablation inductively coupled plasma time-of-flight mass spectrometry (LA-ICP-TOF-MS) imaging is a powerful tool for spatially resolved elemental imaging in heterogeneous tissues. The nematode *Caenorhabditis elegans* (*C. elegans*) is a well-established model organism for metal biology research. However, there have been few reports on mass spectrometry-based metal imaging of *C. elegans* due to challenges in sample preparation for high-resolution and quantitative analysis. In this study, we established an integrated workflow consisting of sample preparation, imaging, and quantitative analysis for whole-animal elemental imaging of *C. elegans* by LA-ICP-TOF-MS. With the assistance of 3D-printed uniform layer media application tools (ULMATs), water-based and hydrocarbon-based embedding media were evaluated, and petrolatum, commercially known as Vaseline, stood out for its performance in preserving *C. elegans* over time for LA-ICP-TOF-MS imaging. Worms were subjected to microscopy and LA-ICP-TOF-MS imaging where we achieved a 2-μm spatial resolution by oversampling laser shots during ablation. Quantitative elemental maps were obtained using gelatin standards sectioned at a 40-μm thickness to closely mimic the average tissue ablation depth of a day 1 adult *C. elegans*. This work established a comprehensive LA-ICP-TOF-MS imaging workflow for model organism *C. elegans* and addressed key analytical bottlenecks associated with sample handling, tissue preservation, spatial resolution, and quantitation in whole-organism elemental imaging of *C. elegans*. The methodology expands the analytical capabilities of LA-ICP-TOF-MS imaging for microscale biological systems and provides an adaptable workflow for future spatial metal biology research using *C. elegans* and other similar model systems.

## Graphical abstract

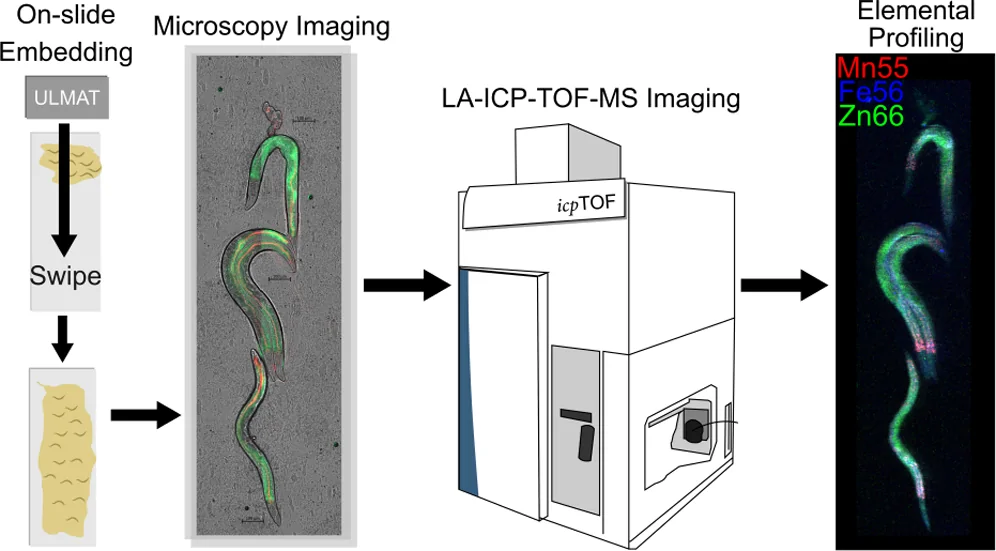

## Introduction

Acquiring, trafficking, and localization of metals in biological systems is essential for physiological processes. Metals can act as structural or catalytic cofactors in cellular pathways such as gene transcription, cell proliferation, and enzymatic reactions.^1,2^ Endogenous metal homeostasis can be altered due to nonessential metal exposure in the environment,^3^ pathogenic infection,^4,5^ autoimmune disorders,^6^ and diseases such as Wilson’s disease and hemochromatosis.^7,8^ Tissues possess multiple cell types resulting in highly heterogeneous systems within an organism. This heterogeneity results in the discrete localization of metal ions, metalloproteins, and metal-binding ligands with respect to individual cell types and their unique biological functions, producing an inorganic phenotype. Excessive exposure to nonessential metals in the environment can disrupt endogenous metal homeostasis and changes in the subcellular distribution and activity of biologically relevant metals.^9^ Alterations in the spatial distribution of metals such as iron in tissues can result in oxidative stress leading to cell death.^10^ Understanding the spatial distribution of metals provides a critical dimension of characterization by relating cell types colocalized with metals *in situ* when investigating metal-related biological processes, and perturbations in metal biology within diseases or disease-states.^11,12^ The importance of contextualizing the spatial distribution of elements in biological systems has resulted in the development and advancement of elemental imaging techniques in recent years to support the growing field of spatial metallomics.^13–16^

A collection of techniques is available to map the spatial distribution of metals in biological samples, including X-ray fluorescence microscopy (XFM), metal-binding fluorescent probes, and mass spectrometry imaging (MSI).^17–19^ While these techniques all seek to quantify and visualize the distribution of elements, each offers advantages and disadvantages. X-ray fluorescence microscopy techniques are nondestructive and offer high spatial resolution imaging (100 nm to 50 μm) with moderate sensitivity and can image large areas of tissue simultaneously. However, XFM struggles with light metal ion imaging.^17^ Furthermore, synchrotron-XFM instrumentation is not readily accessible due to high cost and limited facilities. Metal-binding fluorescent probes serve as another nondestructive metal imaging technique, where fluorophores conjugated with metal chelators change structure upon metal binding and produce optical signals.^20^ While metal-binding fluorescent probes offer good spatial resolution and offers possibilities for live-cell imaging, probes can only bind to free metal ions. Additionally, these probes can be promiscuous and bind to unintended metal ions or adventitious sites which affect accuracy of metal imaging. Finally, laser ablation inductively coupled plasma mass spectrometry (LA-ICP-MS) imaging offers moderate spatial resolution (5–100 μm) with excellent sensitivity, can image large areas of tissue, and is capable of imaging light metal ions.^19^ When coupled with time-of-flight (TOF) mass analyzers, LA-ICP-TOF-MS offers simultaneous detection of most elements on the periodic table in one scan.^21^ While the destructive nature of LA-ICP-MS imaging should be noted, its ability to perform simultaneous detection of multiple elements while balancing detection specificity, sensitivity, and spatial resolution makes it an ideal choice for spatial metallomics.^18^

Many model organisms have been used for the study of metal biology, with the majority of spatial metallomics done on *Daphnia magna*, zebrafish, and mice.^17^ The nematode *Caenorhabditis elegans* (*C. elegans*) is a classical model organism for biology research, offering advantages such as its conserved genetic homology with mammals (60%–80%), tractable genetics, characterizable phenotypes, and ease of culture.^22–24^ *C. elegans* is approximately 1 mm in length at the adult hermaphrodite stage, has distinct tissues and cell types, reproduces rapidly within a short developmental period (approximately 3 days), and possesses many shared mechanisms of metal transport and homeostasis as mammals.^24–26^

Despite the promise of *C. elegans* in metal biology research, limited metal biology research has been done using the organism, with existing research focusing on zinc biology^27^ and metal toxicity.^28^ From existing metal biology research using *C. elegans*, few studies have shown high-spatial resolution metal distributions using LA-ICP-MS imaging, likely due to challenges in sample preparation characterized by desiccation.^29^ An ideal sample preparation method for metal imaging needs to both preserve the worm’s anatomical structures and avoid delocalizing metals. Previous studies relied on sample preparation steps by desiccation or fixation, where desiccation resulted in significant tissue damage.^30–33^ Fixation techniques preserve the worm’s anatomy but can differentially impact metals, thus affecting the localization of metals and introducing interference in detection, particularly for LA-ICP-MS.^34^ Therefore, a method that can preserve the anatomical structure of *C. elegans* without inducing desiccation or tissue damage during handling and avoids the use of harsh chemicals for sample processing is highly desired for spatial metallomic studies using *C. elegans*. In addition, due to the small size of *C. elegans*, high spatial resolution, ideally at cellular levels, is desired to visualize metals in specific tissues for functional interpretations.

In this study, we established an integrated workflow consisting of sample preparation, imaging, and quantitative data analysis for whole-animal elemental imaging of *C. elegans* by LA-ICP-TOF-MS using petrolatum as a preferred embedding medium and achieved 2-μm spatial resolution with laser ablation oversampling. Compared to water-based embedding media, hydrocarbon-based petrolatum showed excellent preservation of *C. elegans* while retaining sufficient optical transparency for microscopy visualization, possessed the necessary nonvolatility for long-term storage, and was elementally pure for high-quality LA-ICP-MS imaging. With the assistance of a home-designed 3D-printed tool, uniform layer media application tool (ULMAT), embedding medium was applied as a uniform layer on top of charged microscope slides for on-slide embedding, freezing, and subsequent elemental analysis of *C. elegans*. An optional and additional step was also used to extract embedded worms from the embedding media layer to prepare petrolatum-coated worms. Comparisons between petrolatum-embedded worms and petrolatum-coated worms were also made to evaluate sample integrity and imaging data quality. Through several time-series experiments at ambient conditions, we evaluated sample integrity under different sample preparation approaches to determine optimal imaging time frames such that tissue decay and metal delocalization did not impede the accuracy of elemental imaging. We then characterized LA-ICP-TOF-MS imaging artifacts indicative of the stages of sample desiccation. We applied an oversampling technique in LA-ICP-TOF-MS imaging and achieved a 2-μm spatial resolution, providing high resolution imaging of *C. elegans* elemental content. Finally, using metal-spiked gelatin standards and ImageJ-based data analysis pipeline, we developed quantitative LA-ICP-TOF-MS imaging workflow and successfully produced quantitative elemental profiling of key biological elements in *C. elegans*.

## Experimental Section

### Materials and Reagents

Optimal cutting temperature compound (OCT), Difco agar, granulated peptone, sodium hydroxide pellets (98%), and Epredia charged microscope slides were purchased from Fisher Scientific (Hanover Park, IL, U.S.A). Gelatin from cold water fish skin, porcine gelatin, carboxymethyl cellulose (CMC, ∼90 000 Da), polyethylene glycol solution (1000 Da, ≥99%) (50% v/v in H_2_O), glycerol (≥99%), sodium chloride (≥99%), calcium chloride dihydrate (≥99%), potassium phosphate monobasic (≥99%), potassium phosphate dibasic (≥99%), magnesium sulfate (≥99%), cholesterol (≥99%), sodium phosphate dibasic (≥99%), and sodium phosphate monobasic (≥99%) were purchased from Sigma-Aldrich (St. Louis, MO, U.S.A). Concentrated Clorox germicidal bleach (8.25% sodium hypochlorite) was purchased from the Michigan State University Stores. Vaseline petroleum jelly (petrolatum) was purchased over the counter. Anycubic Photon Mono 4 and standard resin for 3D printing was purchased from the Anycubic online store. A custom standard IV-74434, 1000 μg/mL (As, B, Ca, Cd, Co, Cr, Cu, Fe, K, Mg, Mn, Mo, Na, Ni, Pb, Se, V, Zn), was purchased from Inorganic Ventures (Christiansburg, VA, U.S.A).

### *Caenorhabditis elegans* Strain Handling

The following *Caenorhabditis elegans* (*C. elegans*) strains were used: N2 (wildtype), and SJ4144 (*zcIs18 [ges-1::GFP­(cyt)]*) which expresses cytosolic GFP in the intestine. SJ4144 was cultured on nematode growth media (NGM) plates at 20 °C fed with *Escherichia coli* (*E. coli)* OP50-tdTomato strain for fluorescence visualization whereas N2 *C. elegans* were cultured on *E. coli* OP50. These strains were purchased from the Caenorhabditis Genetics Center (Minneapolis, MN, U.S.A). To generate large populations of worms for imaging, synchronized SJ4144 *C. elegans* populations were created by isolating embryos from gravid adults using a bleaching solution (0.5 M NaOH, 1.2% NaOCl, 70% H_2_O). Isolated embryos were rinsed with M9 buffer to remove bacteria and incubated overnight.^35^ Once hatched, L1 larvae were transferred to NGM plates seeded with their respective *E. coli* food and allowed to mature. Experiments were performed using day 1 gravid adult worms. To minimize stress and potential metallomic phenotype variation in the synchronized F1 generation from bleach synchronization, wildtype *C. elegans* samples used in quantitative LA-ICP-TOF-MS imaging were synchronized using an egg lay synchronization where 10–20 gravid adults were transferred to seeded NGM plates and allowed to lay eggs in a 3-h period. Gravid adults were removed after the synchronization period, and the remaining eggs were allowed to develop until day 1 gravid adulthood for imaging.

### *C. elegans* Sample Preparation for Elemental Imaging

Day 1 gravid adult *C. elegans* was harvested from the surface of NGM plates by gently washing with M9 buffer and recollecting the worm-containing M9 solution in a microcentrifuge tube. Worms collected this way were centrifuged at 300 g for 1 min, and the supernatant was removed. Fresh M9 buffer was added to the worm pellet, and the rinsing/centrifugation process was repeated at least 3 times or until the supernatant was no longer turbid due to excess bacterial biomass. The remaining pellet was transferred onto an unseeded NGM plate to allow for excess liquid absorption into the NGM agar for 10 min. Once excess liquid was absorbed and worms could freely move on the NGM plate, worms were then used for subsequent on-slide embedding.

### 3D-Printed Uniform Layer Media Application Tool (ULMAT)

A 3D-printed tool made using standard resin, named the uniform layer media application tool (ULMAT), was used to create a uniform ∼100-μm layer of embedding media on top of a glass slide (Figure 1A,B). This tool was printed using an Anycubic Photon Mono 4 resin 3D printer. Essential 3D printing parameters for ULMAT production are listed in Table S1.

**Figure 1:**
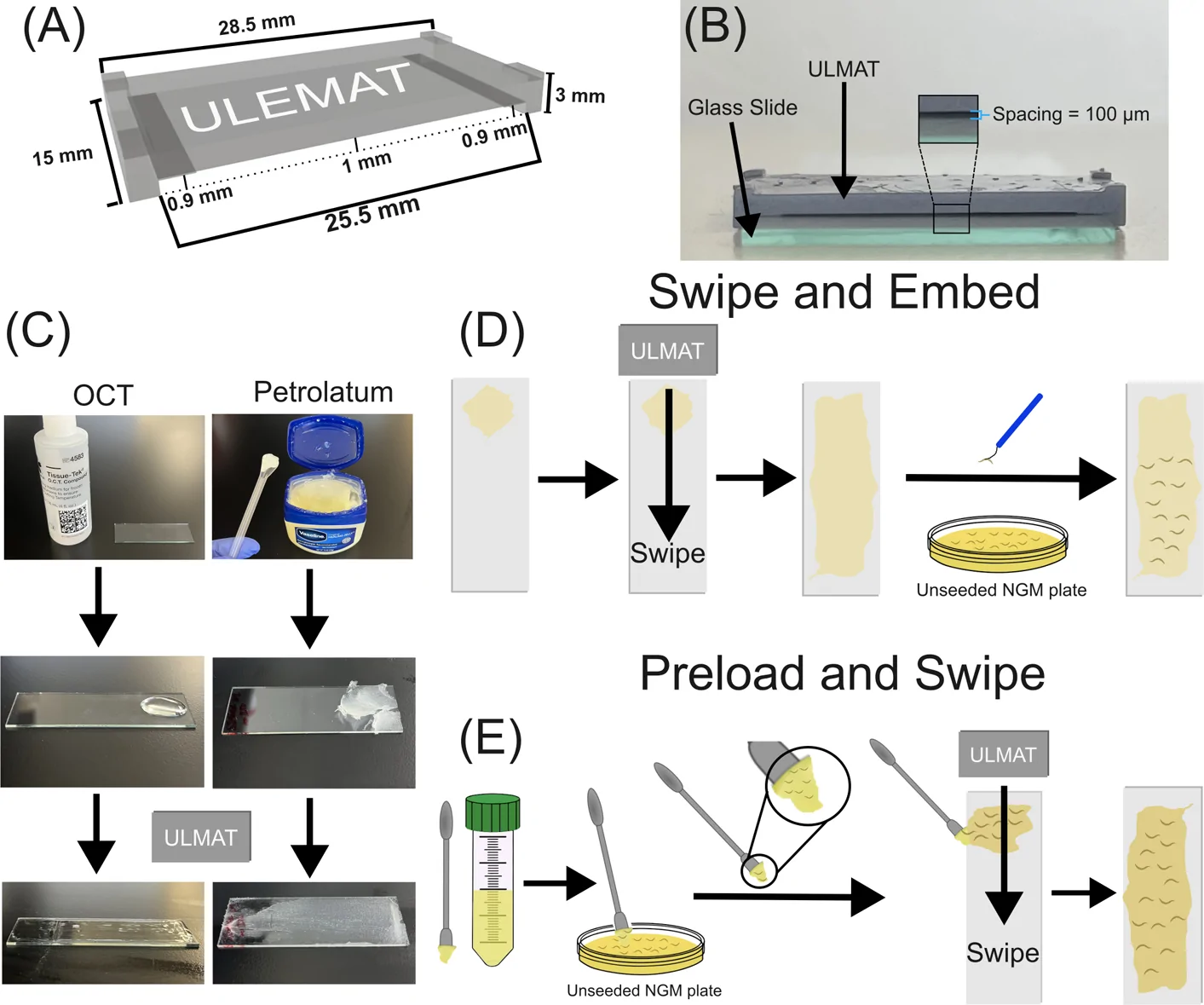
Uniform layer media application tool (ULMAT) design and two ways of application. (A) A transparent 3D design of the ULMAT visualizing the dimensions and unit sizes. (B) A 3D-printed ULMAT mounted directly on top of a microscope slide to visualize the 100-μm spacing for media spreading. (C) Pictures of OCT and petrolatum application and spreading using ULMAT on a microscope slide. (D) Detailed workflow showing how *C. elegans* is transferred into OCT layer after ULMAT application. (E) Detailed workflow showing how *C. elegans* is preloaded into petrolatum by direct transfer and then uniformly spread by ULMAT application.

### On-Slide Embedding of *C. elegans* in OCT and Petrolatum

All sample preparation processes are illustrated in Figure 1 and Figure 2A. ULMATs used for preparing samples were cleaned between batches to prevent sample contamination. This cleaning process consisted of removing excess embedding media from the tool with an ethanol-soaked Kimwipe, followed by a 10-min sonication in a 70% ethanol solution.

#### Swipe-and-Embed

OCT or other water-based embedding medium was added onto the short edge of a glass slide, followed by swiping of the ULMAT tool to form a uniform ∼100-μm layer of OCT on top of the glass. Individual worms prepared on clean NGM plate were transferred using an eyelash tool or a platinum wire pick, followed by gently pressing the worm into the embedding media layer (Figure 1C,D). Embedded *C. elegans* was euthanized by flash-freezing using liquid nitrogen vapors for 3 min and then stored at −80 °C until microscopy and LA-ICP-TOF-MS imaging.

#### Preload-and-Swipe

A plastic spatula was coated in petrolatum and was repeatedly touched across the surface of the NGM plate to pick up 50–100 *C. elegans*. Once the desired number of *C. elegans* was adhered to the petrolatum on the spatula, the petrolatum was transferred to a microscope slide, followed by swiping with the ULMAT to achieve simultaneous on-slide thin layer formation and *C. elegans* embedding (Figure 1C,E). Embedded *C. elegans* was euthanized by flash-freezing using liquid nitrogen vapors for 3 min and then stored at −80 °C until microscopy and LA-ICP-TOF-MS imaging. Worms embedded in petrolatum and frozen this way were placed under vacuum to dry for 5 min to allow water condensate to evaporate from the surface of the embedding media prior to microscopy and LA-ICP-TOF-MS imaging.

#### Petrolatum-Coated

Petrolatum-coated worms were prepared by picking petrolatum-embedded worms with an eyelash pick from the petrolatum layer and transferring them to a new glass slide. These worms were considered “petrolatum-coated” as a small amount of petrolatum remains on the surface of the worm to keep them hydrated.

### Microscopy and LA-ICP-TOF-MS Imaging

After sample processing including embedding, freezing and optional desiccation, brightfield, GFP and tdTomato fluorescence imaging of embedded and coated worms were taken using a Zeiss Axio Imager.Z2 upright fluorescence microscope. Embedded and coated worms were then taken for elemental analysis by LA-ICP-TOF-MS imaging using an ESL Bioimage 266 nm laser ablation system coupled to a TofWerk icpTOF S2. Samples were ablated with a laser power of 90% using a 5-μm spot size and a 3-μm overlap. Imaging cup and imaging chamber flow rates were set to 200 mL/min and 250 mL/min, respectively, using helium gas. Laser repetition rate was set to 125 Hz with a washout time of 10 ms. Plasma, auxiliary, and nebulizer gas flow were set to 14.0 L/min, 8.0 L/min, and 0.9–1.0 L/min, respectively. Measurement mode was kinetic energy discrimination (KED) using a helium gas flow of 5 mL/min. Time-of-flight elemental analysis was done using a *m*/*z* range of 14–256 at a mass resolving power of 1000. All essential microscopy and LA-ICP-TOF-MS parameters for *C. elegans* imaging are shown in Table S2 and Table S3.

### Monitoring *C. elegans* Preservation Postembedding over Time

We first performed monitoring of the same embedded worms over time under a microscope. Petrolatum-embedded worms were frozen by liquid nitrogen vapor for 3 min, thawed, and desiccated at room temperature (20–22 °C) for 5 min to remove excess condensate from the slide. Postprocessing worms were individually imaged and monitored at the following times for brightfield, GFP, and tdTomato microscopy imaging: 1 h, 3 h, 5 h, 8 h, 24 h, and 48 h at room temperature. Petrolatum-coated worms were imaged at the following times for brightfield and GFP imaging: 1 h, 3 h, 5 h, and 8 h at room temperature. These experiments were repeated 3 times using different synchronized populations of SJ4144 to account for differences in preservation quality due to variations in sample preparation and population.

### Investigating Desiccation Effects on Metal Localization in OCT-Embedded *C. elegans*

Day 1 gravid SJ4144 *C. elegans* samples were embedded in OCT, flash-frozen using liquid nitrogen and immediately imaged without the use of gelatin standards. One slide containing SJ4144 *C. elegans* was removed from −80 °C storage and underwent microscopy imaging and LA-ICP-TOF-MS imaging within 1 h at ambient conditions. A second slide from the same population of SJ4144 *C. elegans* was allowed to dry for 1.5 h prior to microscopy imaging and LA-ICP-TOF-MS imaging to compare distribution of elements within imaged worms. Worms were imaged 2–4 h postprocessing this way. Metal distribution was characterized using ^55^Mn as it exhibited the most discrete localization in *C. elegans*, and its subsequent delocalization would be a strong indicator for morphological changes.

### Elemental Quantification of *C. elegans*

To quantify elemental content of wildtype day 1 gravid adult worm samples, 10% (w/w) porcine gelatin standards doped with either a liquid standard mixture (Inorganic Venture custom standard IV-74434) or sodium phosphate monobasic were prepared at a 40-μm thickness. *C. elegans* and standards were scanned at 5-μm spot size and 2-μm raster spacing with a 3-μm overlap to achieve pseudo-2-μm lateral resolution. To eliminate the initial gelatin standard scan which uses the full 5-μm spot size, the imaging run was interrupted after the first line of each gelatin standard was ablated. The initial lines were removed from the defined ROIs such that the requeued imaging run would resume ablating with an effective 2-μm spot size. An aliquot of each gelatin standard was measured by ICP-QQQ-MS to confirm the concentration of each element in each gelatin standard (Table S4). ICP-QQQ-MS parameters for validating gelatin standard concentrations are shown in Table S5. Standards were scanned at equal intervals 10 times for each LA-ICP-TOF-MS run, including at the start and end of the scan. A gas blank was collected for 5 s before each standard scan. Standard concentrations: 0, 12.5, 25, and 50 ppm (IV-74434) and 1000, 2000, and 4000 ppm (sodium phosphate monobasic). All reported ppm concentrations were produced from w/w dilutions and were assumed to be equivalent to μg/g values.

Synchronized populations of N2 *C. elegans* were cultured on *E. coli* OP50 and embedded in OCT, embedded in petrolatum, or coated in petrolatum in separate batches for microscopy assessment and elemental imaging within up to 4-h postprocessing. Ten worms from each population were sampled to compare elemental quantitation results and assess the embedding media matrix effects. These experiments were performed with new gelatin standard sections each day to assess the consistency of quantitation across multiple days of imaging.

### Quantitative Metal Imaging Analysis

Worm dimensions were measured using ImageJ (v1.54p) with the installed macro InteredgeDistance_v1.1_ImageJMacro. Calibrated images were imported into ImageJ, and parallel lines were defined along the body length of *C. elegans* images from the nose to the tail tip. Once lines were defined, the macro randomly sampled between 10 and 50 diameters across the body of the worm between the user-defined lines. The average, standard deviation, number of sampled diameters, minimum measured diameter, and maximum measured diameter were exported into a .csv file for further analysis. The nonuniform rounded shape of *C. elegans* was normalized to a cylinder from the average diameter calculated using ImageJ, with the measured worm length serving as the height of the cylinder. The average depth of tissue ablated by the laser was approximated by redistributing the volume of the cylinder to a cuboid of the same length, where the diameter was retained as the width of the cuboid face. The average depth of tissue ablated across the worm’s body was calculated by dividing the volume of the cuboid by the retained diameter and length (Figure S1). The average depth of tissue ablated was used to determine the appropriate gelatin thickness to best reflect the sample type during quantitative imaging and total metal content in worms.

All LA-ICP-TOF-MS images of *C. elegans* were analyzed and rendered in the Iolite software package.^36^ SJ4144 *C. elegans* images were acquired without the use of gelatin standards for investigations into elemental localization. Generated counts per second (CPS) signals for each element channel were used for elemental map visualization in SJ4144 *C. elegans*. CPS scale bar lower limits were set to 0 and upper limits were set to 99th percentile for ^56^Fe and ^66^Zn whereas ^55^Mn was set to 99.9th percentile. Wildtype *C. elegans* images were acquired with gelatin standards for quantitative imaging. Calibration curves were background-subtracted, and the slopes were forced through the origin (0,0). Limits of detection (LOD) and limits of quantitation (LOQ) were calculated in Iolite as described by Howell et al. (Table S6).^37^ ^31^P quantitation was normalized to the 4000-ppm calibration standard, whereas ^55^Mn, ^56^Fe, ^63^Cu, and ^66^Zn were normalized to the 50-ppm calibration standard. Intensity scale bar lower limits were set to 0 and upper limits were defined for ^55^Mn as 99.9th percentile and 99th percentile for ^31^P, ^56^Fe, ^63^Cu, and ^66^Zn unless otherwise stated. Total metal content in *C. elegans* quantitative imaging was calculated by defining a region of interest (ROI) around *C. elegans* in Iolite and exporting individual pixel concentrations to an Excel workbook. Concentrations in each pixel are reported as parts per million (ppm) and the sample density was assumed to be 1 such that ppm was equivalent to μg/cm^3^. All negative concentration values from pixels were adjusted to values of 0 for total metal content determination. The measured μg/cm^3^ was converted to total μg by multiplying the concentration by the area of the pixel and the average tissue depth in the individual worm. The remaining pixel values were summed to determine total metal content in individual *C. elegans* and converted to total pg due to the low metal content in worms. While this approach underestimates the tissue depth at the center of the worm and overestimates near the edges of the worm, this approach only compromises the spatial accuracy of pg per pixel values. The total pg of the summed pixels remains uncompromised allowing for consistent whole-worm metal content calculations. To account for developmental variations in worm size contributing to metal content differences, total metal content was normalized to the volume of the worm.

### Statistics

Potential embedding media matrix effects were assessed using one-way multivariate ANOVA (MANOVA) for ^55^Mn, ^56^Fe, ^63^Cu, and ^66^Zn in worms. Prerequisite assumption tests for MANOVA (Shapiro-wilk test, Box’s M test) and MANOVA calculations were performed using the python package “statsmodels”. One-way ANOVA was performed on ^55^Mn, ^56^Fe, ^63^Cu, and ^66^Zn after MANOVA to identify which differences among elements contributed to any observed significance identified by MANOVA, with post hoc Bonferroni multiple comparisons test using GraphPad Prism (v10.6.1). Following MANOVA, supervised linear discriminant analysis (LDA) was performed on data to visualize separation among sample preparation approaches. LDA was performed using the python package “scikit-learn” and the samples were split into training (80%, or 22 randomly selected worms) and test (20%, or 6 randomly selected worms) sets. ^31^P content in worms was assessed using one-way ANOVA with Tukey’s HSD post hoc test in GraphPad Prism (v10.6.1) to determine if the washing protocol sufficiently removed the phosphate-containing M9 buffer across all prepared worms.

## Results and Discussion

### A 3D-Printed Tool for Thin-Layer Embedding of *C. elegans* on Glass Slide

To acquire high-resolution LA-ICP-MS images of *C. elegans*, the first key step was to embed the organism in a thin layer of viscous media while maintaining the body in a flat, extended orientation, enabling laser ablation along the worm’s longitudinal axis when sampling. We developed a simple 3D-printed tool to facilitate spreading of a thin and uniform layer of media on a glass slide, named the uniform layer media application tool (ULMAT) (Figure 1A). The ULMAT is a simple tool designed to clasp the edges of a microscope slide while maintaining a gap between the slide surface and the tool (Figure 1A,B). A certain amount of embedding media was transferred to one side of the slide. Embedding media was transferred onto microscope slides and the ULMAT was mounted on top of media and slide. The ULMAT was then moved across the surface of the slide to create a uniform thin layer (Figure 1C). Resin was used to print the tool for a smoother surface texture, as filament-based materials resulted in undesirable streaks on the thin layers of embedding media. For this work, the ULMAT tool was customized to produce reproducible layers of embedding media approximately 100 μm in thickness. This thickness minimizes *C. elegans* out-of-plane orientation as they are typically 50–80 μm at their thickets points depending on their developmental stage while not too thick to impede laser ablation.^38,39^

With the ULMAT tool, we developed two different on-slide embedding approaches for age-synchronized *C. elegans*. The first way, “swipe-and-embed”, is to form a uniform thin layer of media first by swiping (Figure 1C), then transfer individual worms from culture plate to the thin layer using an eyelash tool or a platinum wire pick, followed by gently pressing the worm into the embedding media layer (Figure 1D). This on-slide embedding method gives capability to control the location of transferred worms to facilitate subsequent imaging. While the “swipe-and-embed” method works for hydrophilic media such as OCT, for hydrophobic, semisolid embedding media like petrolatum, it was very challenging to embed worms into the media after the thin layer was already formed. We therefore developed “preload-and-swipe” method by preloading the embedding media with worms before wiping to achieve simultaneous on-slide thin layer formation and *C. elegans* embedding (Figure 1E). The 100-μm layer thickness provides tumbling space to reduce the shear force that worms experience during swiping. This method reduces sample preparation time and preserves the *C. elegans* structure. We also tested “preload-and-swipe” for OCT media and determined it as less optimal as we observed significant worm retention within the pipet tip by surface-tension forces from residual OCT in the tip. Additionally, with the fluid nature of OCT, we found that lateral dispersion of OCT during swiping carried worms to the sides and off the slide which prevented them from being embedded and imaged. Therefore, for subsequent experiments, we applied “swipe-and-embed” to OCT and other aqueous medium and “preload-and-swipe” to the hydrophobic media, petrolatum. After the *C. elegans* embedding, the slide was frozen at −80 °C freezer until next steps.

### Selection of Embedding Media To Preserve *C. elegans* Structural Integrity

Preparation of tissue samples for LA-ICP-MS imaging requires preserving tissue morphology and native distribution of elements over possibly hours of instrumental run time. Traits for an ideal embedding medium should be elementally pure to introduce none-to-little background noise, be optically transparent for microscopy, be capable of preserving *C. elegans* over hours, and not be difficult for the laser to ablate through. We first evaluate the capability of embedding media and application approach to preserve *C. elegans* structural integrity postprocessing (i.e., after embedding, freezing, and optional desiccation) (Figure 2A). Two types of embedding media were tested, including aqueous-based hydrophilic media such as OCT and wax-like hydrophobic media such as petrolatum. An isogenic strain of N2, SJ4144, with GFP fluorescence reporters in intestinal cells, was used to better visualize tissues, and brightfield and fluorescence microscopy were used to evaluate tissue morphology.

**Figure 2:**
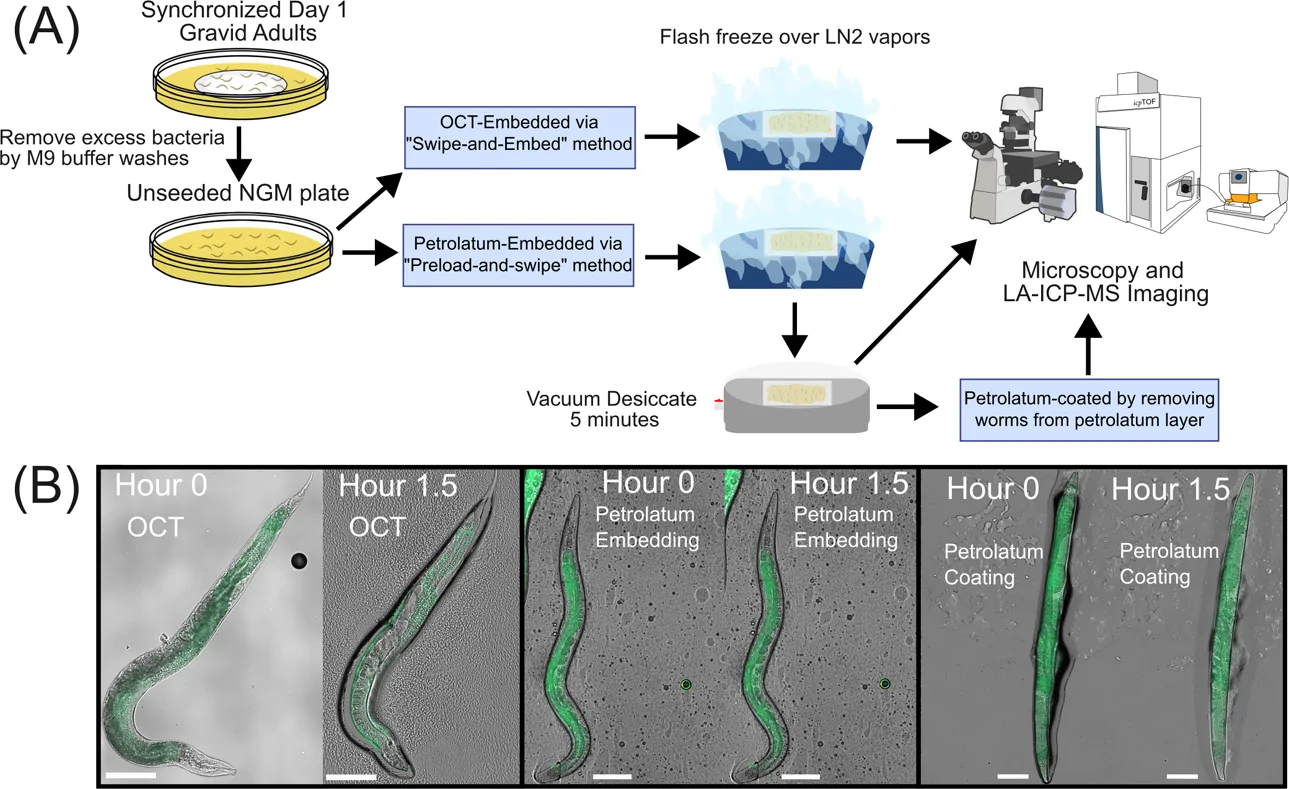
Summary of three *C. elegans* sample preparation and short-term visualization of processed *C. elegans* from different preparation. (A) A combined *C. elegans* embedding workflow for OCT-embedding, petrolatum-embedding, and petrolatum-coating. A synchronized population of day 1 gravid adult *C. elegans* was rinsed with M9 and transferred to an unseeded NGM plate to let the extra M9 buffer absorb into nematode growth media prior to the embedding workflows. (B) Appearance of day 1 gravid adult SJ4144 *C. elegans* embedded in OCT, embedded in petrolatum, and coated in petrolatum observed before and after a 1.5-hour time period, visualizing differences in brightfield, GFP fluorescence, and preservation effectiveness. Brightfield black contrast was set to 0 for all microscopy images. OCT-embedded and petrolatum-coated brightfield white contrast was set to 16 000 and 9000, respectively. Petrolatum-coated brightfield white contrast was set to 6000. Green fluorescence black contrast was set to 150, and white contrast was set to 1500 for all microscopy images. White scale bars in microscopy images represent 100 μm.

We found that the tested water-based embedding media were ineffective in preventing embedded *C. elegans* from desiccation and subsequent structural deformation (Figure 2B, Figure S2). In OCT-embedded worms, the morphology and fluorescence reporter were clearly preserved immediately after the slide was taken out of the freezer (hour 0); however, signs of desiccation appeared after 1.5 h (Figure 2B). Hygroscopic dopants such as glycerol can be added to improve water retention capabilities of these media; however, these molecules typically function as osmolytes^40^ or penetrating cryoprotectants. These additives likely displaced water from tissues, resulting in more rapid tissue desiccation. Compared to OCT, fish gelatin and glycerol-doped CMC showed better preservation of reporter fluorescence and worm body, while the wrinkled appearance of the worm cuticle indicated deformation (Figure S2).

We turned to hydrocarbon-based materials due to their excellent capabilities to maintain moisture in samples. Paraffin is a wax material and used for histology applications but too solid for embedding the soft *C. elegans* body.^41,42^ Petrolatum is another hydrocarbon-based material, that is hydrophobic, nontoxic, nonvolatile, optically transparent, and malleable for embedding at room temperature.^43^ Using the ULMAT tool, we performed on-slide embedding of age-synchronized worms via the “preload-and-swipe” approach (Figure 1E). Petrolatum-embedded worms maintained their morphology and did not show signs of dehydration after being taken out of freezer and sitting at room temperature for 1.5 h (Figure 2B). Major anatomical features can be observed from petrolatum-embedded adult *C. elegans* (Figure S3). During microscopy evaluation, we noticed air bubbles trapped within the petrolatum layer, and we found that before applying petrolatum, heating and sonicating petrolatum in a metal-free tube at 60 °C for 15 min and cooling afterward can effectively degas the media (Figure S4).

During petrolatum embedding via the “preload-and-swipe” approach, we noticed that the worms tend to distribute across the slide in a scattered manner, which resulted in long times spent on searching for worms across the slide to image. We found that petrolatum-embedded worms could be picked up from the petrolatum layer and transferred to a new slide, and the residual petrolatum coated on worm surface was able to keep worms hydrated for durations similar to the petrolatum-embedded worms (“petrolatum coating”, Figure 2). The additional picking and transferring process ensured an operator-controlled layout to reduce the searching time during imaging runs and therefore was kept as a sample preparation method. We noticed that this additional picking step changes the naturally curved posture of an embedded worm to a straight appearance, likely because the embedded worms were flash-frozen but the petrolatum-coated worms were manually manipulated after worms were euthanized (Figure 2B).

To further evaluate worm preservation over time, we extended the postprocessing monitoring period. Petrolatum-embedded worms showed extended preservation over at least 8 h postprocessing (Figure S5). Decay of GFP reporter (starting after 3 h), increase of nonspecific fluorescence (starting after 24 h), and deformation of body and pharyngeal morphology were progressively observed over the course of 48 h (Figures S5 and S6), indicative of worm death and decay that can be possibly attributed to rigor mortis and intestinal necrosis.^44^ We also noticed minimal fluorescence of the tdTomato reporter from *E. coli* OP50 food between 0 and 24 h postprocessing, indicating that our washing protocol effectively removed intestinal contents. Monitoring petrolatum-coated worms over 8 h using microscopy showed that the worms progressively dehydrated over 8 h; thus, structural preservation from petrolatum-coating preparation was not as good as petrolatum-embedding (Figure S7). We noticed that petrolatum coating resulted in diffused fluorescence signals compared to petrolatum embedding, making it challenging to recognize reporter-labeled tissues, possibly due to the GFP fluorescent light emitted from the intestinal cells undergoing diffuse reflection in the uneven petrolatum coating the surface of the worm.^45,46^ Altogether, our results showed that OCT and petrolatum can preserve *C. elegans* structure for varying amounts of time, with petrolatum capable of preserving worm morphology for at least 3 h, significantly longer than OCT, and both media were subsequently evaluated with LA-ICP-MS imaging.

### Impact of Laser Parameters and Sample Preparation on Worm LA-ICP-TOF-MS Imaging

Due to the small size of *C. elegans* (∼1 mm in length, ∼50–80 μm in diameter), it is essential to tune laser parameters to maximize signal and spatial resolution. LA-ICP-MS imaging typically use spatial resolutions of 5–50 μm, which provides adequate spatial resolution for larger tissues from model organisms such as rats, mice, zebrafish, and *Daphnia magna*.^47–49^ To improve spatial resolution for *C. elegans*, we utilized an oversampling strategy to raster the ablation laser of 5-μm spot size with a 2-μm step size, thus achieving an effective 2-μm spot size.^50,51^ To ensure that worms were completely ablated during imaging, elemental signatures unique to glass were monitored during imaging runs with this oversampling method. LA-ICP-TOF-MS imaging of worms showed ^27^Al signal produced from alumina (Al_2_O_3_) in glass across the imaged area from a single pass. This confirmed that samples were completely ablated during LA-ICP-TOF-MS imaging (Figure S8). We found that the effective 2-μm spot size via oversampling also provided sufficient spatial resolution for visualization of ^55^Mn between paired intestinal cells, which could not be achieved by using a 5-μm laser profile with no oversampling, showing the importance of high-resolution imaging for *C. elegans* (Figure S9).

To evaluate impact of sample preparation on LA-ICP-TOF-MS imaging results, we performed oversampled 2-μm imaging and used ^55^Mn as a benchmark to compare results from different preparations (Figure 3). Initial microscopy evaluation indicated preservation of *C. elegans* morphology, anatomy, and fluorescence at *T* = 0 (Figure 3A). At 60-min postprocessing, OCT-embedded *C. elegans* experienced some level of metal delocalization; specifically, the ^55^Mn signals were more diffused compared to petrolatum preserved samples (Figure 3B). The distribution of ^55^Mn in OCT-embedded *C. elegans* appeared to be altered compared to existing synchrotron-XFM literature,^52^ resulting in large paired patterns between intestinal cells in many worms. Both petrolatum-embedded and -coated *C. elegans* showed clear, highly localized ^55^Mn signals in the intestine. Occasionally, incomplete ablation was observed due to uneven embedding in petrolatum (Figure S10), but the frequency was low. Picking out worms from petrolatum to generate petrolatum-coated worms can reduce the chance of having unevenly embedded worms and ensure that laser is mostly ablating worm tissue instead of embedding media but adds an additional user step.

**Figure 3:**
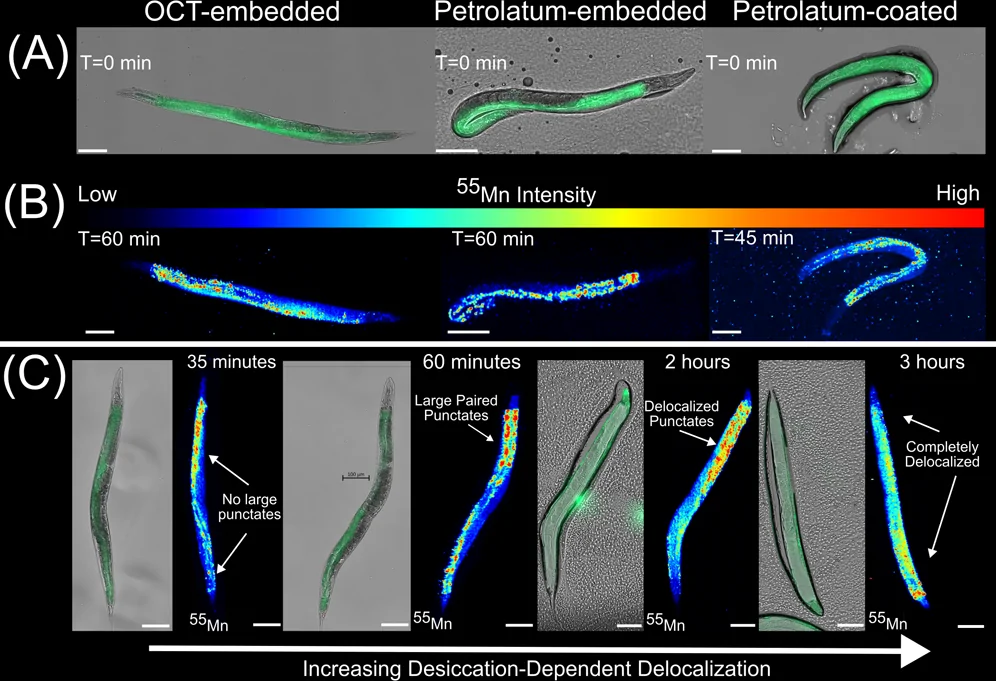
Investigating elemental distribution in *C. elegans* from different sample preparation techniques. (A) Microscopy imaging of day 1 gravid adult SJ4144 *C. elegans* prepared by OCT-embedding, petrolatum-embedding, and petrolatum-coating at *T* = 0. Microscopy images were taken shortly after removing samples from −80 °C storage and designated as *T* = 0 as the starting point for ambient condition. (B) LA-ICP-TOF-MS images visualizing ^55^Mn. LA-ICP-TOF-MS imaging of worms was completed within 60 min, with their completion times listed in the corresponding ^55^Mn ion images. (C) ^55^Mn delocalization in OCT-embedded *C. elegans* was characterized at hours 0–4 at ambient conditions. Ideal ^55^Mn localization was found along the intestinal lumen, with progressive delocalization observed with respect to time and desiccation progression within the laser ablation stage. ^55^Mn ion intensities were extracted as counts per second, with the lower limit set to 0th percentile and the upper limit set to 99.9th percentile for all ion images. White scale bars in microscopy images and LA-ICP-TOF-MS ion images represent 100 μm.

We speculated that the metal delocalization in OCT-embedded worms was due to desiccation and thus further tested whether desiccation causes artifacts on metal distribution analysis using ^55^Mn as a benchmark (Figure 3C). Results confirmed that OCT-embedded *C. elegans* experienced morphological changes over time shown by microscopy and LA-ICP-TOF-MS imaging, likely due to rigor mortis changing the worm orientation or desiccation. ^55^Mn in existing synchrotron XFM imaging shows discrete localization along the intestinal lumen,^52^ which can be observed in OCT-embedded *C. elegans* samples that were imaged by LA-ICP-TOF-MS under short imaging times. However, OCT-embedded worms sitting for longer times at ambient conditions postprocessing induced gradual delocalization resulting in total delocalization of metals by 3 h, further emphasizing that OCT is not suitable for long-term preservation of *C. elegans*.

### Simultaneous Mapping of Multiple Elements by High-Resolution LA-ICP-TOF-MS

The LA-ICP-TOF-MS technology provides the capability to image most elements on the periodic table simultaneously with the TOF analyzer. Using this technology, we successfully demonstrated simultaneous mapping of biologically relevant elements such as ^31^P, ^55^Mn, ^56^Fe, ^63^Cu, and ^66^Zn in *C. elegans*, exceeding previous quadrupole-based LA-ICP-MS studies on *C. elegans* with one or two elements and their respective isotopes.^31–33^ The metallome of *C. elegans* consists of Na, Mg, K, Ca, Mn, Fe, Cu, and Zn representing the most abundant metals, and lower-abundance metals such as Co, Mo, and Se.^53–56^ While LA-ICP-TOF-MS as a technology provided simultaneous visualization of all relevant elements in analyzed *C. elegans*, in this work, we present the five most abundant biologically relevant elements, particularly for later quantitative analysis, for their biological importance.

We visualize ^31^P, ^55^Mn, ^56^Fe, ^63^Cu, and ^66^Zn simultaneously in day 1 gravid adult *C. elegans* prepared by both petrolatum coating (Figure 4A) and petrolatum embedding (Figure 4B). By comparing elemental images with brightfield microscope images, we were able to observe discrete visualization of *C. elegans* anatomy for the assignment of elemental localization. In petrolatum-coated worms, ^31^P was found throughout the worm’s body and most strongly accumulated in the intestinal cells and posterior region of the worm. ^31^P is an essential element in biomolecules such as DNA, phospholipids, energy-storage molecules such as ATP, and as such, its abundance can reflect the cell size and activity across regions of tissue.^57^ ^55^Mn exhibited the most discrete localization of imaged elements throughout the intestinal tract of *C. elegans* with a punctate pattern. This defined distribution of ^55^Mn has previously only been visualized using synchrotron-XFM technology and was first achieved in this work with LA-ICP-MS imaging.^52^ ^56^Fe showed some increased background noise due to the presence of the isobaric interference ArO^+^; however, we used kinetic energy discrimination within the collision cell and improved ^56^Fe signals. ^56^Fe and ^55^Mn showed higher relative abundances in the anterior intestine in likely the grinder, with significant localization to the intestinal cells throughout the worm body. Meanwhile, ^56^Fe did not exhibit a punctate distribution like ^55^Mn throughout intestinal cells. While an essential element, ^63^Cu was found in low abundance throughout *C. elegans*, potentially due to the organism’s reduced use of the element in biological processes.^58^ Improved imaging of ^63^Cu can be achieved by using LA-ICP-QQQ-MS imaging if copper biology is of interest. While ^55^Mn and ^56^Fe showed enrichment in *C. elegans*’ anterior intestine, ^66^Zn showed little or no enrichment in the anterior region of *C. elegans* and instead showed increased abundance in the posterior region of imaged worms. Synchrotron-XFM imaging has revealed similar zinc distributions with posterior intestinal cells on day 5 *C. elegans* possessing high zinc content;^52^ however, our 2-μm spatial resolution is not sufficient to resolve the zinc-rich gonad arms from the intestine. Additionally, the orientation of *C. elegans* during imaging may confound resolving the gonad arms from the intestine. We also noticed that images from petrolatum-coated worms showed a noisier background compared to others (Figures 2B and 4A). One likely reason is that the laser was directly ablating the bare glass slides without embedding media on top of it and therefore introduced more interfering signals from glass slides contaminants such as ^56^Fe from iron oxide (Fe_2_O_3_) and ^66^Zn from zinc oxide (ZnO). Additionally, metal contamination could be introduced during the transfer step in creating petrolatum-coated worms and increased background noises. Metal impurities may be found in adhesives such as glue,^59,60^ which was used to construct the eyelash tool for transferring worms. Repeated cleaning of the eyelash pick between transfers using 70% ethanol solutions may result in a slow dissolution of the glue, which may have subsequently resulted in leaching of metal impurities onto the glass slide when dragged across the surface during transfer. Further investigations should be made to examine possible sources of metal contamination during sample preparation.

**Figure 4:**
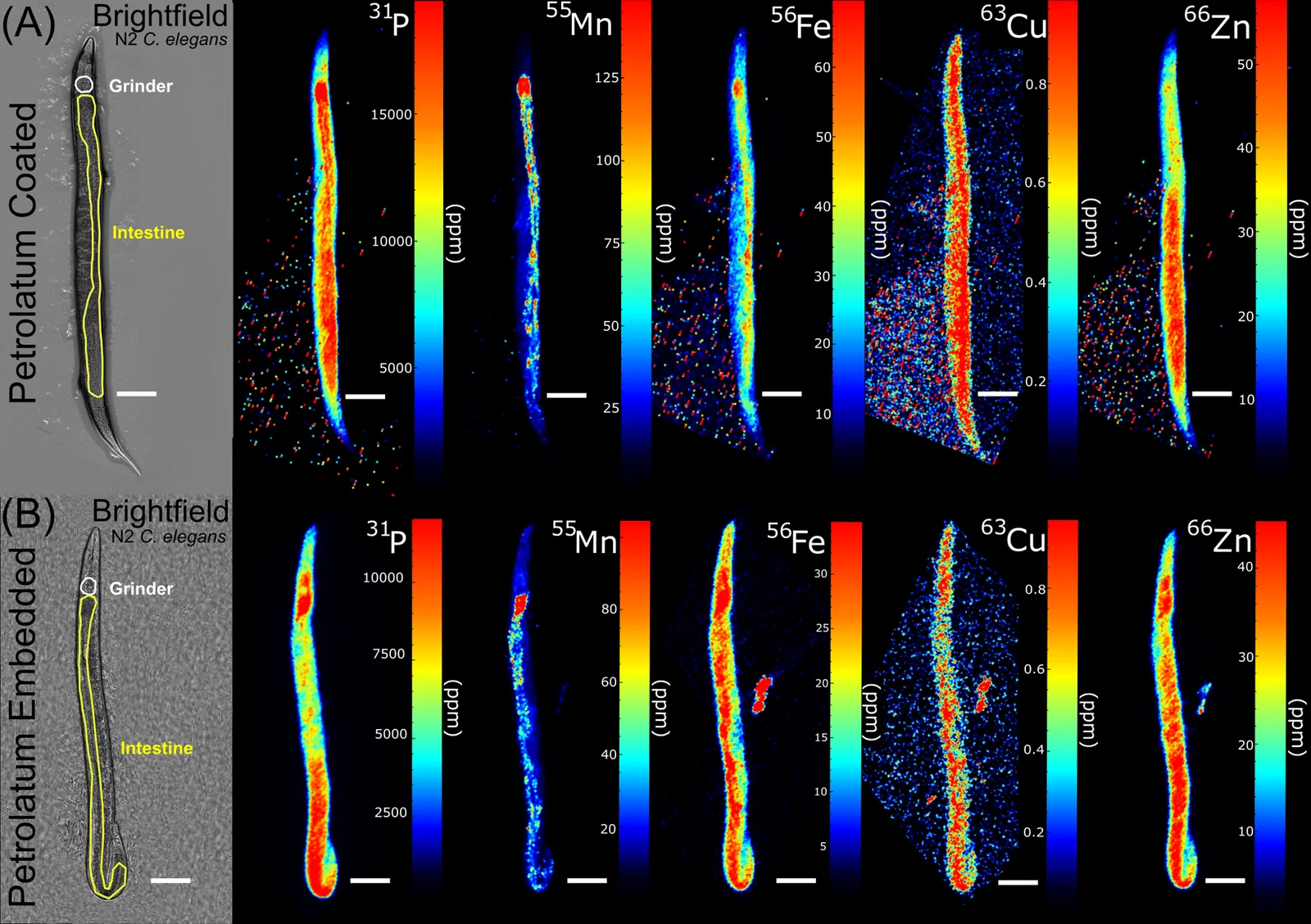
Brightfield microscopy and elemental imaging of day 1 gravid adult wildtype N2 *C. elegans* prepared by petrolatum-based methods. (A) Brightfield microscopy and LA-ICP-TOF-MS imaging of a petrolatum-coated *C. elegans.* (B) Brightfield microscopy and LA-ICP-TOF-MS imaging of a petrolatum-embedded *C. elegans*. LA-ICP-TOF-MS imaging was able to perform simultaneous mapping of ^31^P and essential trace elements including ^55^Mn, ^56^Fe, ^63^Cu, and ^66^Zn. Lower limits for all elements were set to 0 ppm, and upper limits were set to 99th percentile for all elements except ^55^Mn which was set to 99.9th percentile. Brightfield black contrast and white contrast were set to 0 and 9000, respectively. White scale bars represent 100 μm.

When comparing petrolatum-coated elemental images (Figure 4A) to petrolatum-embedded elemental images (Figure 4B), several notable differences were observed. The background signal was drastically reduced in all ion images of petrolatum-embedded *C. elegans*. Elemental contaminants are present in glass slides, and with petrolatum serving as a barrier between the laser and the microscope slide, these contaminant signals are not observed through the direct ablation of glass. Additionally, minor differences in elemental localization were observed between petrolatum-embedded and -coated *C. elegans*. Across petrolatum-embedded replicates, elements that were found to be primarily intestinally localized in petrolatum-coated images showed somewhat broadened localization such as ^31^P and ^56^Fe. We also observed differences in concentration scaling for ^31^P, ^55^Mn, ^56^Fe, ^63^Cu, and ^66^Zn across imaged replicates. Such variation could be attributed to embedding media matrix effects altering plume properties and impacting spatial clarity in imaging or could be due to biological variations. Future studies should focus on characterizing the impact of worm orientation, embedding media thickness, and developmental variations on the slight differences in metal distribution.

### Metal Quantification Pipeline with Calibration Curves from LA-ICP-TOF-MS Analysis

We further developed a quantification pipeline to quantify the distribution and total amount of elements from LA-ICP-TOF-MS imaging. External calibration curves are commonly used for LA-ICP analysis by using standard-doped gelatin pads of certain thickness to mimic tissue.^61,62^ Unlike cryosectioned animal tissues with the same thickness across tissue, *C. elegans* possesses a tapered, cylindrical body shape with uneven thickness across its length and width, which complicates the quantification from imaging because the laser will ablate different thicknesses across its body. Therefore, the first step was to choose a thickness of standard-doped gelatin pads that can represent *C. elegans* volume. Approximations of *C. elegans*’ dimensions have been reported previously by treating *C. elegans*’ shape as a cylinder.^63^ We further approximate the cylinder to a rectangular cuboid to obtain a uniform thickness by matching the *C. elegans* body volume and length such that standards of adequate thickness could be produced for quantitative imaging (Figure S1). Through ImageJ analysis, we determined the average diameter and length of the body of an early day 1 gravid adult (1–3 h into adulthood) wildtype *C. elegans* to be 52.12 μm and 933.02 μm, respectively (Figure S11A,B). These values generally agree with existing studies on *C. elegans* growth dynamics, suggesting that the ImageJ macro produces accurate measurements of *C. elegans*.^63,64^ The average tissue ablation depth across the entire body of *C. elegans* from this cylinder normalization was furthermore found to be 40.93 μm (Figure S11C). From these measurements, the volume of *C. elegans* could be calculated for further insight into how metal content correlates with growth dynamics or metal-content normalization (Figure S11D). As gelatin is the most conventional standard material for animal tissue for LA-ICP-TOF-MS imaging,^61,62^ from the calculated average tissue ablation depth, we determined that sectioning gelatin standards doped with element standards at a 40-μm thickness would sufficiently reflect the sample type’s average thickness.

We investigated interday and intraday variations of calibration curves as part of the quantitative LA-ICP-TOF-MS imaging experiments for worms. Calibration curves produced from raw data showed noticeable interday variations in sensitivity over a total of 8 imaging experiments, while calibration curves generally produced satisfactory *R* ^2^ values (>0.95) (Figure S12). Investigations into individual imaging runs showed intraday drifting of ion intensity and sensitivity (Figure S13). Generally, the signal intensity and calibration curve slopes decreased in sensitivity as an imaging run progressed. Factors such as temperature changes may also affect signal acquisition by reducing detector sensitivity, efficiency of vaporization, ablated particle size, and ionization.^65,66^ Future work may explore postacquisition correction of possible instrumental drift with external software such as AutoSpect.^67^ Limits of detection (LODs) and limits of quantitation (LOQs) from interday imaging experiments showed large relative standard deviation percentages (RSD%) (Table S6). Despite that, there was no significant impact on elemental quantitation as all LODs and LOQs fell below the lowest gelatin standard concentration (12.5 ppm for ^55^Mn, ^56^Fe, ^63^Cu, ^66^Zn; 1000 ppm for ^31^P) except for one imaging experiment where the LOQ for ^31^P was measured as 1278 ppm (Table S6).

### Evaluating Embedding Media Matrix Effects through Single Worm Metal Quantification

Single-worm metal contents from quantitative LA-ICP-TOF-MS imaging were determined to assess matrix effects in OCT-embedded, petrolatum-embedded, and petrolatum-coated worms. Single-worm quantification has typically reported values of metals in the picogram per worm range.^68^ However, *C. elegans* experiences rapid growth and development throughout adulthood,^69^ which can result in size-dependent differences in the total metal content. Normalizing to phosphorus or sulfur content is a common approach to improving data comparability between biological replicates but is not suitable for investigating matrix effects between groups,^70^ as matrix effects can unilaterally affect all signals acquired on the same sample, including phosphorus and sulfur, such that total metal content may change but the ratio of the analyte ion and normalization ion is unchanged. Ultimately, a normalization strategy to account for biological variation using a feature that is independent of matrix effects is desired to allow for the evaluation of embedding media matrix effects among three sample preparation techniques. Therefore, we opted to normalize total metal contents, in both mass and molarity, from individual *C. elegans* to the respective volume of the analyzed individual.

Unnormalized total element content, volume-normalized total element content, and molar concentrations of metals from OCT-embedded, petrolatum-embedded, and petrolatum-coated *C. elegans* are shown in Table 1. ^31^P is the only measured element where components of the washing buffer may interfere with reproducibility. M9 buffer does not contain transition metal salts and only contains potassium, sodium, and magnesium salts. Among these salts, sodium phosphate dibasic and potassium phosphate monobasic exist at millimolar concentrations which can confound phosphorus quantitation from rinsing variations. Among the 8 repeated experiments for washing and imaging *C. elegans*, we noticed that 6 imaged worms from 2 days from the petrolatum-coated group showed significantly elevated ^31^P content (Figure S14). These worms were prepared on high humidity days which prevented adequate evaporation and absorption of the washing buffer into NGM plates from the surface of the worms. When these 2 days are excluded from ^31^P quantitation comparisons, the adjusted ^31^P content produces comparable values to ^31^P from OCT-embedded and petrolatum-embedded groups (Table 1). Future LA-ICP-TOF-MS imaging of *C. elegans* should use phosphate-free buffers to wash worms of bacteria and not perturb ^31^P quantitation. Unnormalized essential transition metal content between sample preparation groups also varied significantly; when normalized to respective worm volume, these adjusted values reduced metal content differences due to developmental variation for better comparison between groups (Table 1, Figure S15). Elemental content variance generally fell between 20% and 45% percent relative standard deviation in both un-normalized and normalized data sets. To our knowledge, this is the first report in the quantitative metal profiles of day 1 gravid adult *C. elegans*, and the variance reported in these data may be confounded by the developmental transition from young adult to early gravid adult. Future research should seek to investigate how *C. elegans’* metallome evolves from early adulthood to later stages of adulthood to better contextualize these data and the observed variance to biological variance.

**Table 1:** Metal Content per Worm Measured by LA-ICP-TOF-MS Imaging^a^

sample preparation	element	total content, pg	total content normalized to volume,^a^ pg/μm^3^	total content normalized to volume,^a^ μmol/L	replicates
OCT-embedded	^31^P	9.14 × 10^3^ (16%)	4.53 × 10^–3^ (23%)	1.46 × 10^5^ (23%)	*n* = 10
	^55^Mn	18.5 (35%)	9.13 × 10^–6^ (37%)	166 (37%)	*n* = 10
	^56^Fe	39.6 (33%)	1.96 × 10^–5^ (33%)	350 (33%)	*n* = 10
	^63^Cu	0.945 (33%)	4.69 × 10^–7^ (36%)	7.45 (36%)	*n* = 10
	^66^Zn	39.1 (33%)	1.93 × 10^–5^ (32%)	292 (32%)	*n* = 10
petrolatum-embedded	^31^P	1.70 × 10^4^ (38%)	8.80 × 10^–3^ (33%)	2.85 × 10^5^ (33%)	*n* = 8^b^
	^55^Mn	29.3 (24%)	1.55 × 10^–5^ (36%)	299 (36%)	*n* = 8
	^56^Fe	38.2 (21%)	2.01 × 10^–5^ (24%)	376 (24%)	*n* = 8
	^63^Cu	1.60 (45%)	8.35 × 10^–7^ (41%)	13.7 (41%)	*n* = 8
	^66^Zn	50.2 (18%)	2.65 × 10^–5^ (24%)	421 (24%)	*n* = 8
petrolatum-coated	^31^P	8.02 × 10^4^ (78%)	3.75 × 10^–2^ (90%)^c^	1.21 × 10^6^ (90%)^c^	*n* = 10
		2.07 × 10^4^ (15%)	6.67 × 10^–3^ (13%)^d^	2.15 × 10^5^ (13%)^d^	*n* = 4
	^55^Mn	43.6 (48%)	1.68 × 10^–5^ (31%)	305 (31%)	*n* = 10
	^56^Fe	53.8 (16%)	2.23 × 10^–5^ (22%)	397 (22%)	*n* = 10
	^63^Cu	2.87 (35%)	1.20 × 10^–6^ (40%)	19.1 (40%)	*n* = 10
	^66^Zn	71.8 (30%)	2.95 × 10^–5^ (32%)	446 (32%)	*n* = 10

a Elemental content per worm. Values shown as average (RSD%).

b Measured metal contents in LA-ICP-TOF-MS imaged worms were normalized to respective worm body volume.

c Two worms were not completely ablated and omitted from total element content calculations.

d Phosphorus content altered due to possible residual M9 buffer on imaged worms from humid working conditions.

e Subset of worms after excluding worms presumably had residual M9 due to high humidity of preparation day.

One-way multivariate ANOVA (MANOVA) was used to assess differences in elemental profiles by simultaneously considering all dependent variables during statistical analysis. Through this statistical test, the collective variation of ^55^Mn, ^56^Fe, ^63^Cu, and ^66^Zn was analyzed with respect to sample preparation technique. This approach eliminates the risk of family-wise type I error rate inflation that would occur through repeated one-way ANOVA of each dependent variable.^71^ MANOVA analysis showed statistically significant differences in total metal content among preparation techniques (Wilks’ Λ = 0.2429, *F*(8, 44) = 5.659, *p* < 0.001). One-way ANOVA with post hoc Bonferroni multiple comparisons was performed on ^55^Mn, ^56^Fe, ^63^Cu, and ^66^Zn concentrations to determine which elements contributed most to elemental profile differences (Figure 5A), showing that ^55^Mn, ^63^Cu, and ^66^Zn concentrations from OCT-embedded worms were significantly different from petrolatum-coated worms. For petrolatum-embedded worms, only ^55^Mn was statistically significantly different from OCT-embedded worms. Following MANOVA, linear discriminant analysis (LDA) revealed that both petrolatum-based methods clustered similarly, indicating that these two groups did not separate from each other. Interestingly, a multivariate outlier appeared in the petrolatum-coated *C. elegans* group that did not appear as an outlier from preliminary univariate investigations (Figure 5B). Evaluation of the LDA model determined the macro-AUC of the training and test splits as 0.957 and 0.963, suggesting that the model performed well, and that the observed outlier is a product of extreme biological variance (Figure S16). LDA also revealed that the OCT-embedded group clustered separately and had the least overlap with confidence regions from the two petrolatum-embedded groups (Figure 5B), corroborating the MANOVA analysis.

**Figure 5:**
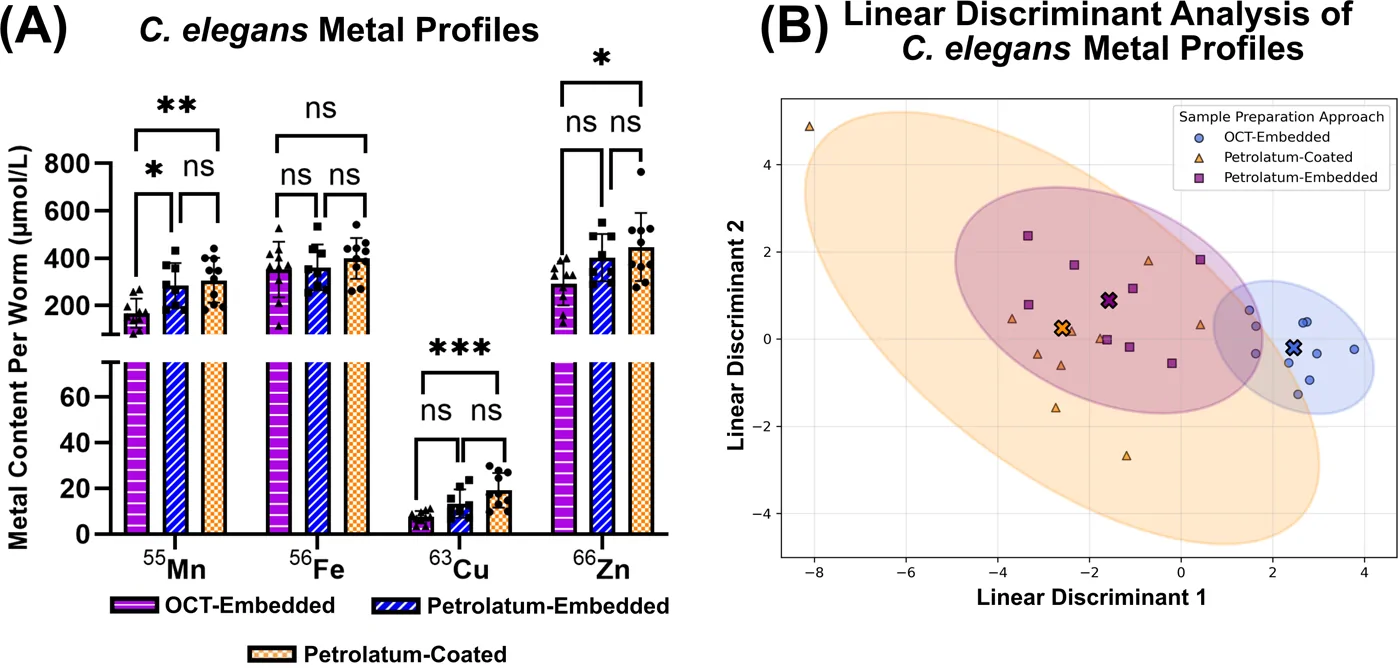
Statistical analysis of total metal content in *C. elegans* from quantitative LA-ICP-TOF-MS imaging prepared using different embedding techniques. (A) Volume-normalized metal content from *C. elegans* visualizing statistically significant differences in metal content between sample preparation techniques from MANOVA, ANOVA, and post hoc testing. Different colored and patterned bars represent the total metal content of worms for ^55^Mn, ^56^Fe, ^63^Cu, and ^66^Zn. Solid shapes indicate individual replicates (*n* = 10 for OCT-embedded, *n* = 8 for petrolatum-embedded, *n* = 10 for petrolatum-coated worms). One-way MANOVA (Wilks’ Λ = 0.2429, *F*(8, 44) = 5.659, *p* < 0.001) showed statistically significant differences in total metal content among preparation techniques. One-way ANOVA with Bonferroni multiple comparisons test determined that ^55^Mn, ^63^Cu, and ^66^Zn contributed to observed significant differences. (B) Linear discriminant analysis (LDA) of z-score normalized *C. elegans* metal profiles visualizing both petrolatum-based methods cluster similarly while OCT-embedded *C. elegans* showed least overlap. OCT-embedded data points are represented by blue circles, petrolatum-embedded data points are represented by purple squares, and petrolatum-coated are represented by orange triangles. Colored ellipses represent 95% confidence regions, while correspondingly colored X’s indicating group centroids. * = *p* < 0.05.

When quantified using gelatin standards, OCT-embedded *C. elegans* yielded the least signal for all elements when compared to both petrolatum-based methods (Wilks’ Λ = 0.2429, *F*(8, 44) = 5.659, *p* < 0.001), indicating that the residual OCT film on dried *C. elegans* may suppress plume formation during laser ablation. While no statistical significance between the two petrolatum-based methods was observed, previous imaging results showed that petrolatum-embedded *C. elegans* could have inconsistent ablation whereas petrolatum-coated *C. elegans* did not experience this (Figure S10).

### Summary Comparisons of Three On-Slide Embedding Preparations

Each sample preparation approach for LA-ICP-TOF-MS imaging of *C. elegans* offers different advantages and limitations and is summarized graphically in Figure 6. OCT-embedded and petrolatum are commercially available and elementally pure for quantitative elemental imaging applications. OCT-embedded offers the highest quality microscopy imaging with minimal nonspecific fluorescence, however, as a water-based embedding media, it desiccates rapidly, which impacts *C. elegans* morphology and metal localization. Petrolatum coating offers consistent ablation with moderate sample preservation qualities, allowing for preservation up to 8 h at ambient conditions. Nonspecific fluorescence remains an issue with the petrolatum-coating method, which prevents multimodal imaging applications using fluorescence microscopy. Petrolatum embedding offers the longest sample preservation of the explored workflows and generates moderate nonspecific fluorescence to enable the application of tissue-specific fluorescence reporters, making it desirable for metal-tissue colocalization analysis. However, petrolatum embedding risks occasional insufficient ablation due to the thickness of the petrolatum layer.

**Figure 6:**
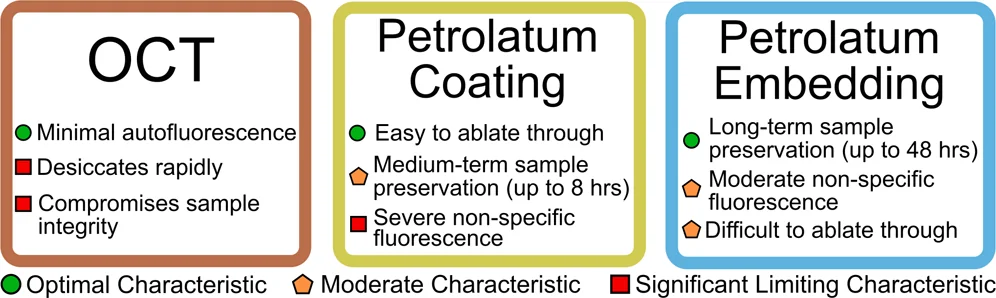
Summary of three tested embedding approaches for microscopy and LA-ICP-TOF-MS imaging of *C. elegans*. Characteristics of a specific preparation technique are listed within the colored bordered boxes. Green circles indicate the characteristic is highly desirable, orange pentagons indicate that the characteristic is not inherently optimal but manageable and acceptable, and red squares indicate that the characteristic is a significant limiting factor that needs careful experimental design to optimize the preparation technique.

## Conclusion and Limitations

LA-ICP-MS imaging is an important imaging modality for metal biology research in model animals. Previous studies have performed LA-ICP-MS imaging on *C. elegans*, but the sample preparation and technological limitations made it challenging to achieve high-resolution, multi-element, and quantitative imaging.^31–33^ We developed an integrated workflow that includes sample preparation, instrumental analysis and data processing to enable high-resolution LA-ICP-TOF-MS imaging with quantification on *C. elegans*, providing a powerful tool for future metal biology research in this classical biology model. We first developed a 3D-printed tool to create a uniform and a thin layer of viscous embedding media on glass slide for on-slide embedding of *C. elegans*. Three different sample preparations, OCT-embedded, petrolatum-embedded and petrolatum-coated, were evaluated using microscopy and subsequent LA-ICP-MS imaging. We demonstrated that petrolatum preserved reporter fluorescence, tissue morphology, and elemental distribution for a longer period when compared to conventional embedding media such as OCT. Through oversampling laser shots, we were able to achieve an effective 2-μm spatial resolution in LA-ICP-MS imaging, allowing for resolving cellular elemental compartmentalization that has been previously only observed in XFM studies.^52,72^ We further established a data analysis pipeline toward quantitative LA-ICP-TOF-MS imaging. With gelatin standards, we demonstrated quantitative imaging of five endogenous elements simultaneously in one sample, allowing colocalization of worm fluorescence reporters, brightfield images and different elements.

There are several limitations of this study that will be addressed in future research. First, the gelatin standards may not fully reflect the nature of *C. elegans* tissue. Future work should consider other types of media to recapitulate the interaction between *C. elegans* tissue and laser ablation for a more accurate quantitative LA-ICP-MS analysis. In several cases, extrapolation of the gelatin standard calibration curve was needed to accommodate metal concentration dynamic ranges in *C. elegans* such as ^55^Mn in the instance of high per pixel elemental concentrations. Future work will adjust concentration ranges to encompass elemental concentrations observed in *C. elegans* without extrapolation. Additionally, in this work, we focused on five elements, particularly for quantitative analysis, instead of a full panel of metals in *C. elegans* for LA-ICP-MS imaging. Future work should explore further optimization of the parameters to fully utilize the potential of the TOF analyzer to simultaneously detect more elements in one sample. Lastly, XFM studies on day 1 worms may be considered to serve as a ground truth of quantitative elemental distribution.

## Supplementary Material





## Data Availability

The data that support the findings of this study are available in the methods, results, and supplementary material of this article. Processed data, calculations, and STL file for the ULMAT used in this study can be found at https://doi.org/10.6084/m9.figshare.33442729. Other original data sets are available upon request to the corresponding authors.

## Notes

Detailed instrument parameters for LA-ICP-TOF-MS, ICP-QQQ-MS, microscopy, and 3D-printing, measured concentrations of gelatin standards used for quantitative LA-ICP-TOF-MS imaging, evaluation of instrument performance for quantitative LA-ICP-TOF-MS imaging, additional characterization of petrolatum-based embedding and imaging of worms, analysis of *C. elegans* dimensions, comparison of different laser raster patterns and imaging resolution, identification of elemental signatures to confirm complete sample ablation, additional elemental quantitation of *C. elegans* by quantitative LA-ICP-TOF-MS imaging, evaluation of LDA model performance (PDF)
Raw data of *C. elegans* dimension quantification using ImageJ (XLSX)
