# Supplementary material for: On-Slide Preparation of Caenorhabditis elegans toward Quantitative, High-Resolution LA-ICP-TOF Mass Spectrometry Imaging
Source: Anal Chem. 2026 Sep 17;98(38):28440–53. doi: 10.1021/acs.analchem.6c04271 (PMC13629169; doi:10.1021/acs.analchem.6c04271)
Supplement: Supplementary file 1 [file ac6c04271_si_001.pdf]

Supporting Information

On-slide Preparation of *Caenorhabditis elegans* Towards Quantitative, High-Resolution LA-ICP-TOF Mass Spectrometry Imaging

Aidan J. Reynolds[1#], Aaron Sue[2,3#], Keith MacRenaris[2,3], Thomas V. O'Halloran[1,2,3*], Tian (Autumn) Qiu[1*]

[1]Department of Chemistry, Michigan State University, East Lansing, MI, 48824, USA

[2]Department of Microbiology, Genetics and Immunology, Michigan State University, East Lansing, MI, 48824, USA

[3]Elemental Health Institute, Michigan State University, East Lansing, MI 48824, USA

*Co-corresponding authors: Tian (Autumn) Qiu, qiutian8@msu.edu; Thomas V. O'Halloran, ohallor8@msu.edu.

[#]A.J.R. and A.S. are co-first authors of this work.

Table of Contents

Table S1: Anycubic Photon Mono 4 3D printing parameters
Table S2: Microscopy imaging parameters
Table S3: LA-ICP-TOF imaging parameters
Table S4: Measured Gelatin Standard Concentrations
Table S5: ICP-QQQ-MS Parameters
Table S6: Calculated limits of detection and quantitation
Figure S1: *C. elegans* dimension analysis and workflow for determining appropriate gelatin standard thickness
Figure S2: Microscopy imaging of *C. elegans* embedded in alternative media
Figure S3: Anatomical feature assignment to petrolatum-embedded *C. elegans*
Figure S4: Bubbles in petrolatum
Figure S5: Petrolatum-embedded worms observation, time series
Figure S6: Changes in *C. elegans* morphology due to intestinal necrosis
Figure S7: Petrolatum-coated microscopy time-series imaging
Figure S8: Elemental indicators present in microscope slides for complete sample ablation
Figure S9: 5-μm versus 2-μm spatial resolution LA-ICP-TOF-MS imaging
Figure S10: Variation in *C. elegans* embedding height in petrolatum may cause inconsistent LA-ICP-TOF-MS imaging
Figure S11: Dimensions of *C. elegans* determined by ImageJ
Figure S12: Multi-day calibration curve data
Figure S13: Calibration Curve sensitivity drift within one experiment
Figure S14: Evaluation of residual ^{31}P content from M9 buffer rinsing
Figure S15: Unnormalized versus volume-normalized total metal content in *C. elegans*
Figure S16: Macro-average ROC curves evaluating LDA model performance

Supporting Information

Table S1: Anycubic Photon Mono 4 3D Printer Parameters

Slice Parameters			
Layers Thickness (mm)	0.050	Control Type	Basic
Normal Exposure Time (s)	2.5	Z Lift Distance (mm)	6
Off Time (s)	1	Z Lift Speed (mm/s)	4
Bottom Exposure Time (s)	30	Z Retract Speed (mm/s)	6
Bottom Layers	5		
Anti-alias	1		
Use Random Erode Shell	Off		

Table S2: Zeiss Axio Imager.Z2 Microscopy imaging parameters

Brightfield parameters			***GFP channel parameters***			***mCherry/tdTomato channel parameters***		
**Parameter**	**Unit**	**Value**	**Parameter**	**Unit**	**Value**	**Parameter**	**Unit**	**Value**
Lightsource intensity (TL Halogen lamp)	V	4.2	Lightsource intensity (Xcite Xylis lamp)	%	15	Lightsource intensity (Xcite Xylis lamp)	%	15
Exposure time	ms	10.00	Exposure time	ms	100	Exposure time	ms	100
Intensity	%	30	Intensity	%	15	Intensity	%	30
Black contrast	~	0	Black contrast	~	200	Black contrast	~	150
White contrast	~	6000-9000	White contrast	~	1500	White contrast	~	750
Camera parameters								
**Parameter**	**Unit**	**Value**						
Exposure time	ms	10.00						
Auto Exposure	on/off	on						
Intensity	%	30						

Table S3: Essential LA-ICP-TOF MS imaging parameters

ICP-MS Parameters (Tofwerk S2)			***Time-of-Flight Parameters (Tofwerk S2)***			**Laser Ablation Parameters (ESL Bioimage 266 nm)**		
**Parameter**	**Unit**	**Value**	**Parameter**	**Unit**	**Value**	**Parameter**	**Unit**	**Value**
**RF Power**	W	1550	**m/z range**	amu	14 - 256	**Spot Size**	μm	5
**Sampling Depth**	mm	4.9	**Resolution**	m/Δm	1000	**Interline Distance (y)**	μm	2
**Cone Material**	Nickel	-	**ODG Settings**	ms	20	**Overlap (x)**	μm	3
**Cone Insert (STD)**	mm	3.5	**Notch Bias**	V	-80	**Repetition Rate**	Hz	125
**Plasma Gas Flow**	L/min	14.0	**Notch (40 amu)**	V	3	**Laser Power**	%	70
**Auxillary Gas Flow**	L/min	8.0	**Notch (36 amu)**	V	1	**Laser Fluence**	J/cm^2	9.5-10.2
**Nebulizer Gas Flow**	L/min	0.9 - 1.0	**Notch (28 amu)**	V	2	**Sample Energy**	mJ	.005
**Measurement Mode**	CCT Mode	-	**Notch (15.8 amu)**	V	3	**Imaging Cup Flow Rate (He)**	mL/min	200
**CCT Gas Flow (100% He)**	mL/min	5				**Imaging Chamber Flow Rate (He)**	mL/min	250
**CCT Focus lens**	V	-19.5				**PEEK Tubing I.D.**	mm	0.75
**CCT Entry Lens**	V	-180				**Washout Time**	ms	10
**CCT Mass**	V	250						
**CCT Bias**	V	-9						
**CCT Exit Lens**	V	-200						

Table S4: Essential element concentrations in gelatin standards measured by ICP-QQQ-MS

Standard	^{31}P	^{55}Mn	^{56}Fe	^{63}Cu	^{66}Zn
Gelatin 50 ppm	~	50.18	51.50	50.59	50.45
Gelatin 25 ppm	~	24.06	24.30	23.49	23.47
Gelatin 12.5 ppm	~	11.37	11.73	11.18	11.61
Gelatin 4000 ppm	3798.42	~	~	~	~
Gelatin 2000 ppm	1870.44	~	~	~	~
Gelatin 1000 ppm	945.54	~	~	~	~
Gelatin Blank	3.29	0.02	0.56	0.026	0.74

Table S5: Essential ICP-QQQ-MS parameters

ICP-MS Parameters (Agilent 8900 ICP-QQQ-MS)					
**Parameter**	**Unit**	**Value**	**Parameter**	**Unit**	**Value**
**RF Power**	W	1550	**Cell Focus**	-V	-4.0
**Sampling Depth**	mm	8.0	**Cell Entrance**	V	-50
**Cone Material**	Nickel	-	**Cell Exit**	V	-70
**Plasma Gas Flow**	L/min	15.0	**Deflect**	V	-4.2
**Auxillary Gas Flow**	L/min	0.9	**Plate Bias**	V	-60
**Nebulizer Gas Flow**	L/min	1.09	**OctP Bias**	V	-18.0
**Measurement Mode**	CCT Mode	-	**OctP RF**	V	180
**CCT Gas Flow (100% He)**	mL/min	5	**Energy Discrimination**	V	3.0
**Extraction Lense 1**	V	-12.0			
**Extraction Lense 2**	V	-210.0			
**Omega Bias**	V	-130			
**Omega Lens**	V	8.6			
**Q1 Entrance**	V	-50.0			
**Q1 Exit**	V	2.0			

Table S6: Quantitative LA-ICP-TOF-MS Limits of Detection and Quantitation

Calculated LODs and LOQs reported as parts per million (ppm)							
			Element				
Imaging Experiment			^{31}P	^{55}Mn	^{56}Fe	^{63}Cu	^{66}Zn
OCT-Embedded	1	LOD	11.44	0.04	0.41	0.04	0.13
		LOQ	34.32	0.12	1.22	0.13	0.38
OCT-Embedded	2	LOD	20.03	0.03	0.19	0.03	0.07
		LOQ	60.10	0.10	0.57	0.08	0.21
Petrolatum-Embedded	3	LOD	17.19	0.10	0.47	0.08	0.33
		LOQ	51.57	0.29	1.42	0.24	0.99
Petrolatum-Embedded	4	LOD	49.47	0.06	1.42	0.11	3.38
		LOQ	148.41	0.19	4.26	0.34	10.14
Petrolatum-Embedded	5	LOD	118.11	0.09	0.36	0.07	0.29
		LOQ	354.32	0.26	1.08	0.20	0.87
Petrolatum-Coated	6	LOD	42.06	0.06	0.56	0.07	0.16
		LOQ	126.18	0.19	1.67	0.20	0.48
Petrolatum-Coated	7	LOD	426.04	0.11	1.33	0.25	0.81
		LOQ	1278.12	0.34	4.00	0.74	2.42
Petrolatum-Coated	8	LOD	9.46	0.06	0.39	0.05	0.17
		LOQ	28.37	0.17	1.17	0.14	0.51
Averages (RSD%)		LOD	86.72 (163.3%)	0.07 (40.1%)	0.64 (72.7%)	0.09 (81.8%)	0.67 (167.8%)
		LOQ	260.17 (163.3%)	0.21 (40.1%)	1.92 (72.7%)	0.26 (81.8%)	2.00 (167.8%)

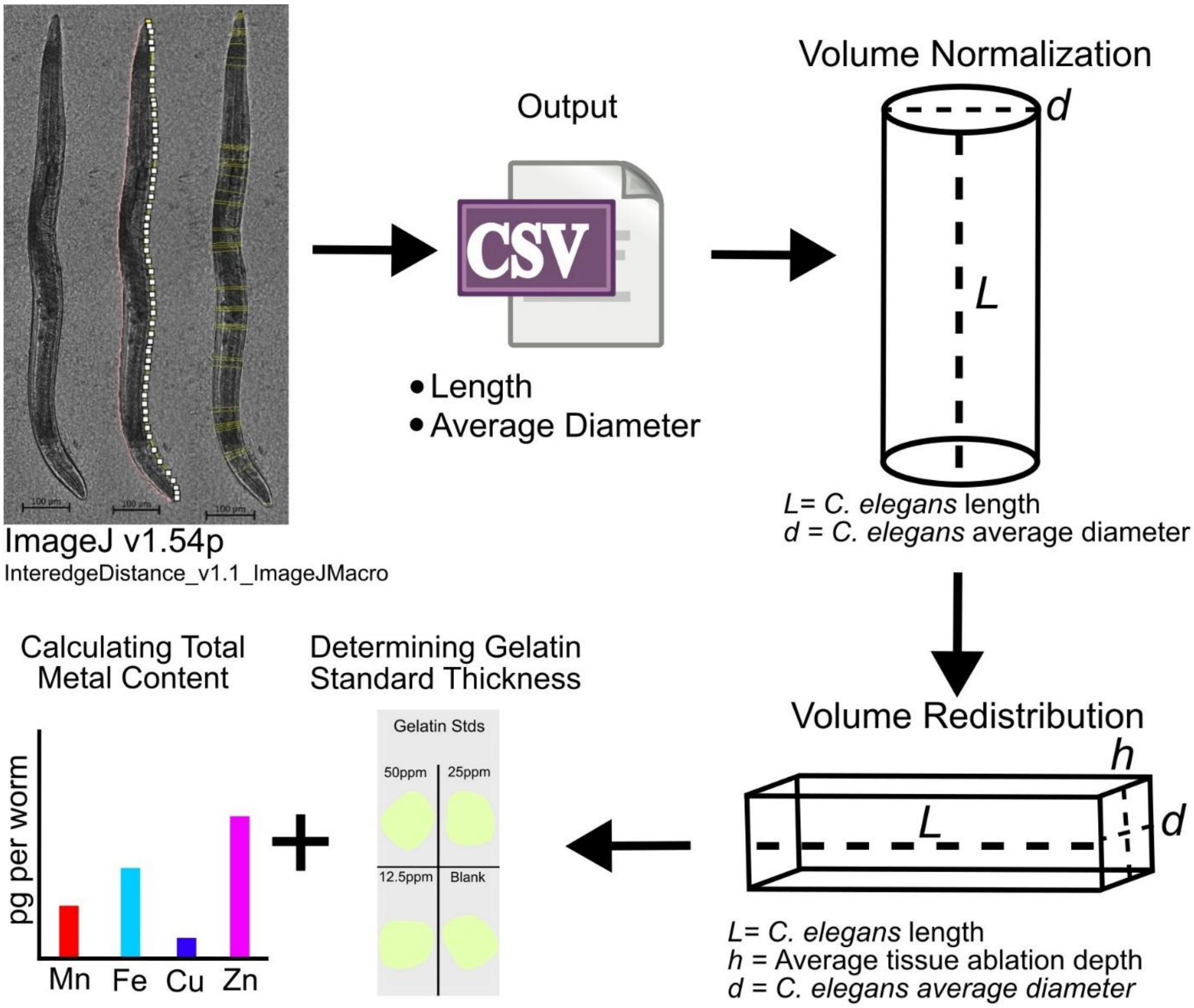

Figure S1: Workflow diagram for determining the dimensions of imaged *C. elegans* using ImageJ and the macro InteredgeDistance_v1.1_ImageJMacro. Microscopy images of worms are calibrated and imported into ImageJ where the user defines the outline of the sample along the left and right sides of the worm. The length of the worm is measured, and the diameter of the worm is sampled at up to 50 different positions along the user defined lines, generating an average diameter. *C. elegans* volume is determined from the measured lengths and diameters and the volume of the worm is normalized to the shape of cylinder. The volume is then redistributed from a cylinder to a cuboid to determine the average thickness of the worm. The average thickness of the worm is then used for downstream processes including gelatin standard thickness determination and total metal content extraction from LA-ICP-TOF-MS images.

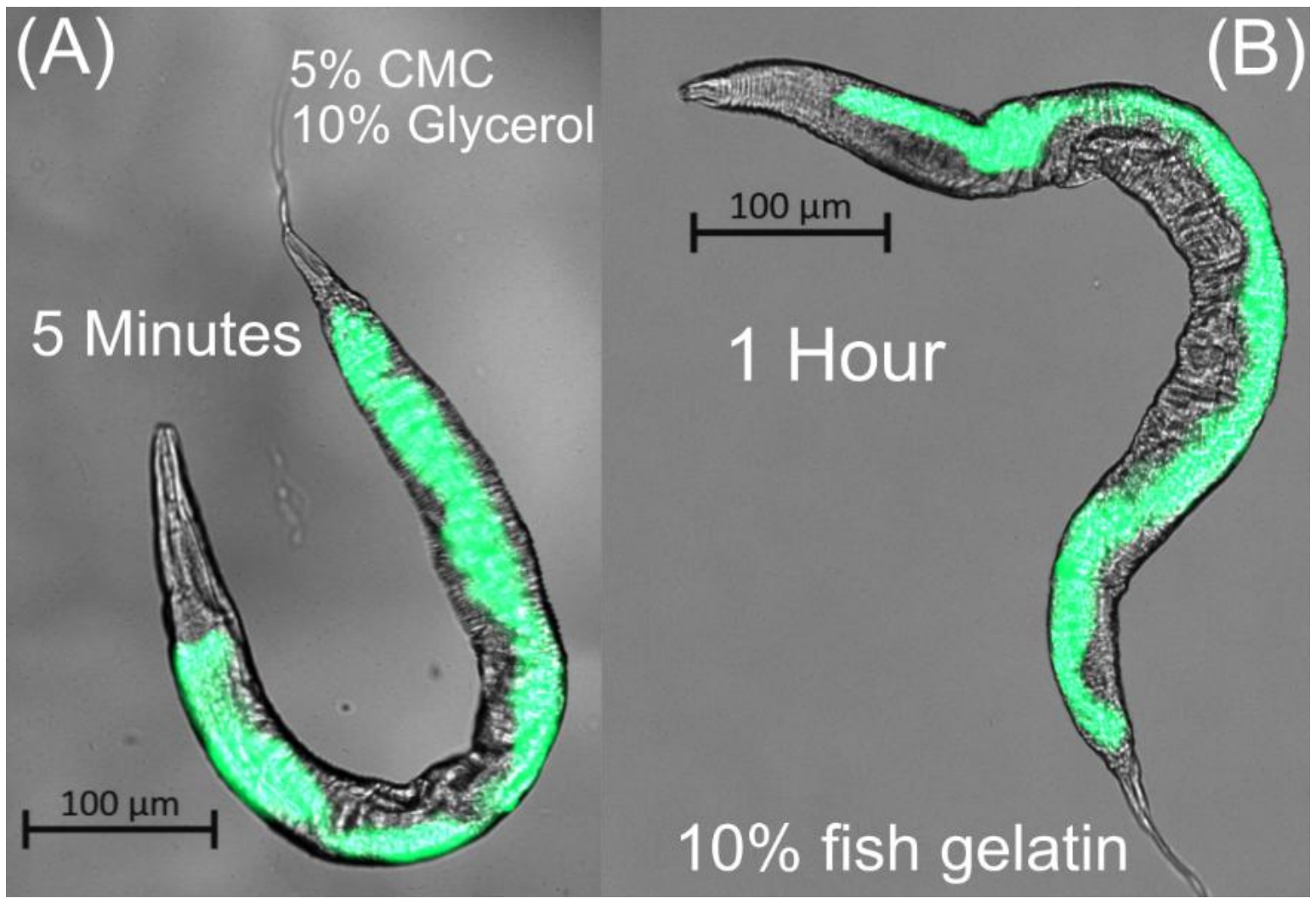

Figure S2: Brightfield and GFP imaging of a gravid adult day 1 SJ4144 *C.* elegans embedded in alternative water-based embedding media. (A) A day 1 gravid SJ4144 *C.* elegans in 5% carboxymethyl cellulose (CMC) spiked with 10% glycerol. (B) A day 1 gravid SJ4144 *C.* elegans in 10% fish gelatin. GFP expression visualizes the intestine of *C. elegans*. The wrinkled cuticle in the brightfield channel indicates desiccation. Times indicate the earliest point when complete desiccation occurred. Black scale bars indicate 100 µm.

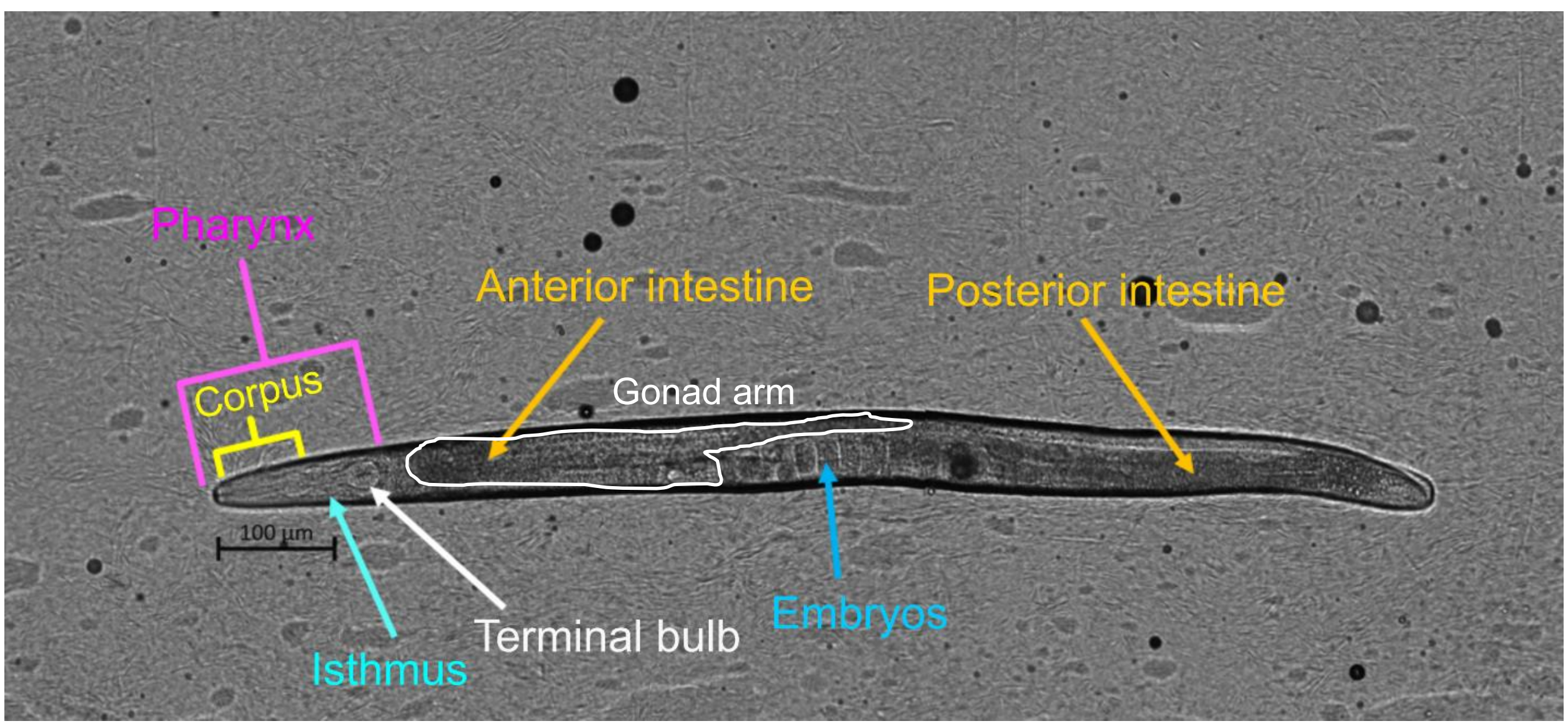

Figure S3: Identification of major anatomical features in an adult hermaphroditic *C. elegans* embedded in petrolatum. The pharynx is identified by the pink brackets with pharyngeal components labeled with the white, cyan and yellow indicators. The intestinal tract is identified by the orange indicators and embryos are identified in the blue indicator. Black scale bar indicate 100 µm.

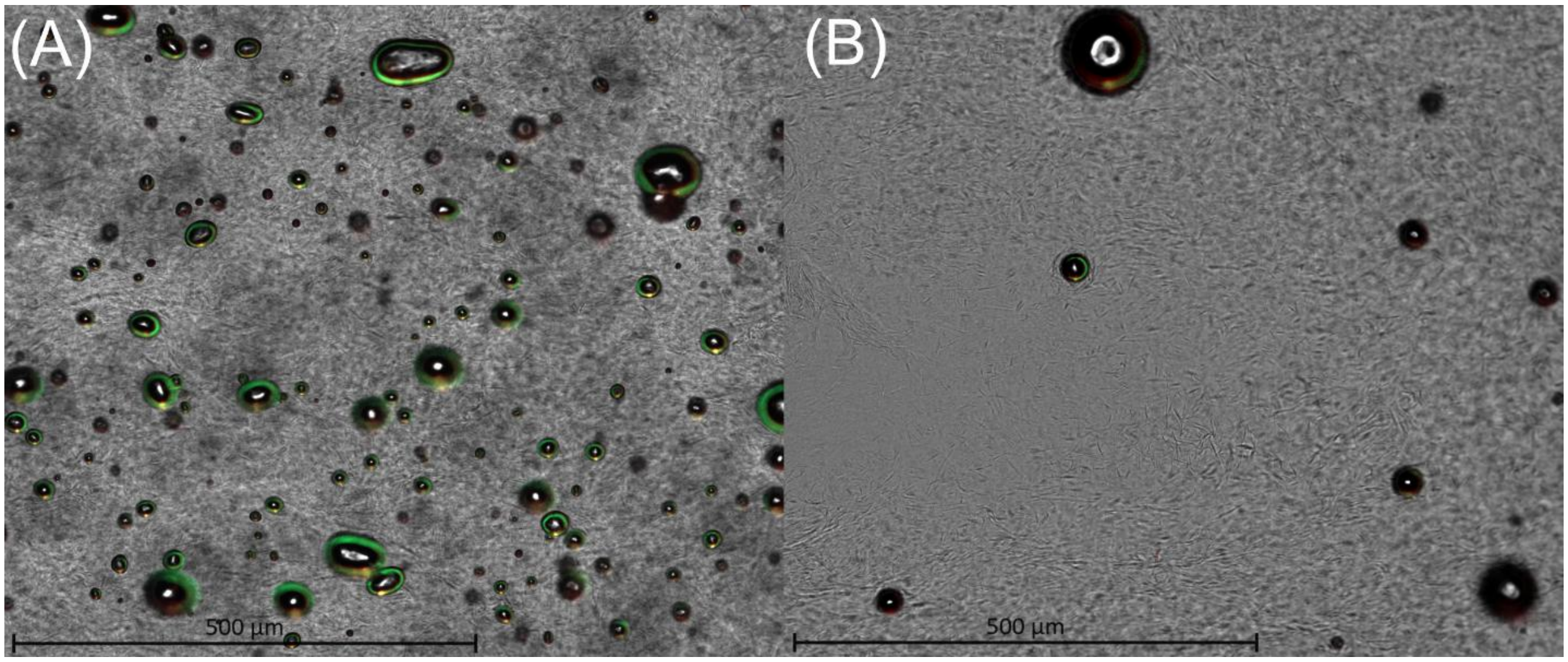

Figure S4: Petrolatum characteristics viewed under microscope. (A) Initial petrolatum application directly from Vaseline container, (B) and pre-heated and degassed petrolatum application showing that heating and sonicating petrolatum prior to application can reduce bubble formation during the embedded process. Black scale bars indicate 500 μm.

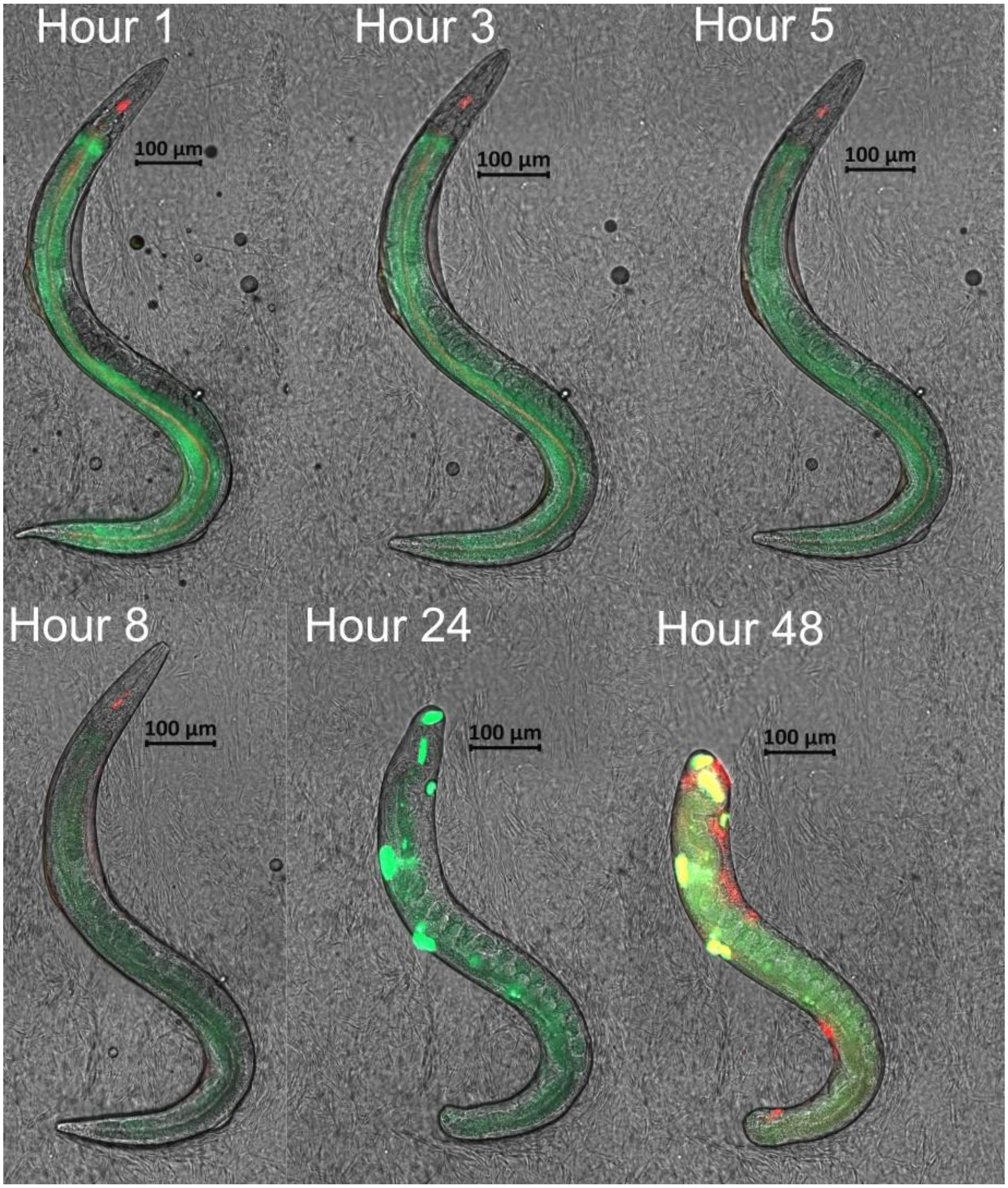

Figure S5: Evaluation of petrolatum for day 1 gravid adult SJ4144 *C. elegans* preservation and 48-hr tracking on post-processing preservation at ambient conditions. Petrolatum-embedded worms were sufficiently preserved such that tissue decay occurred prior to desiccation. Green channel fluorescence imaging reveals intestinally expressed cytosolic GFP. Red channel fluorescence imaging reveals the localization of tdTomato from bacterial matter within *C. elegans*. Black scale bars indicate 100 µm.

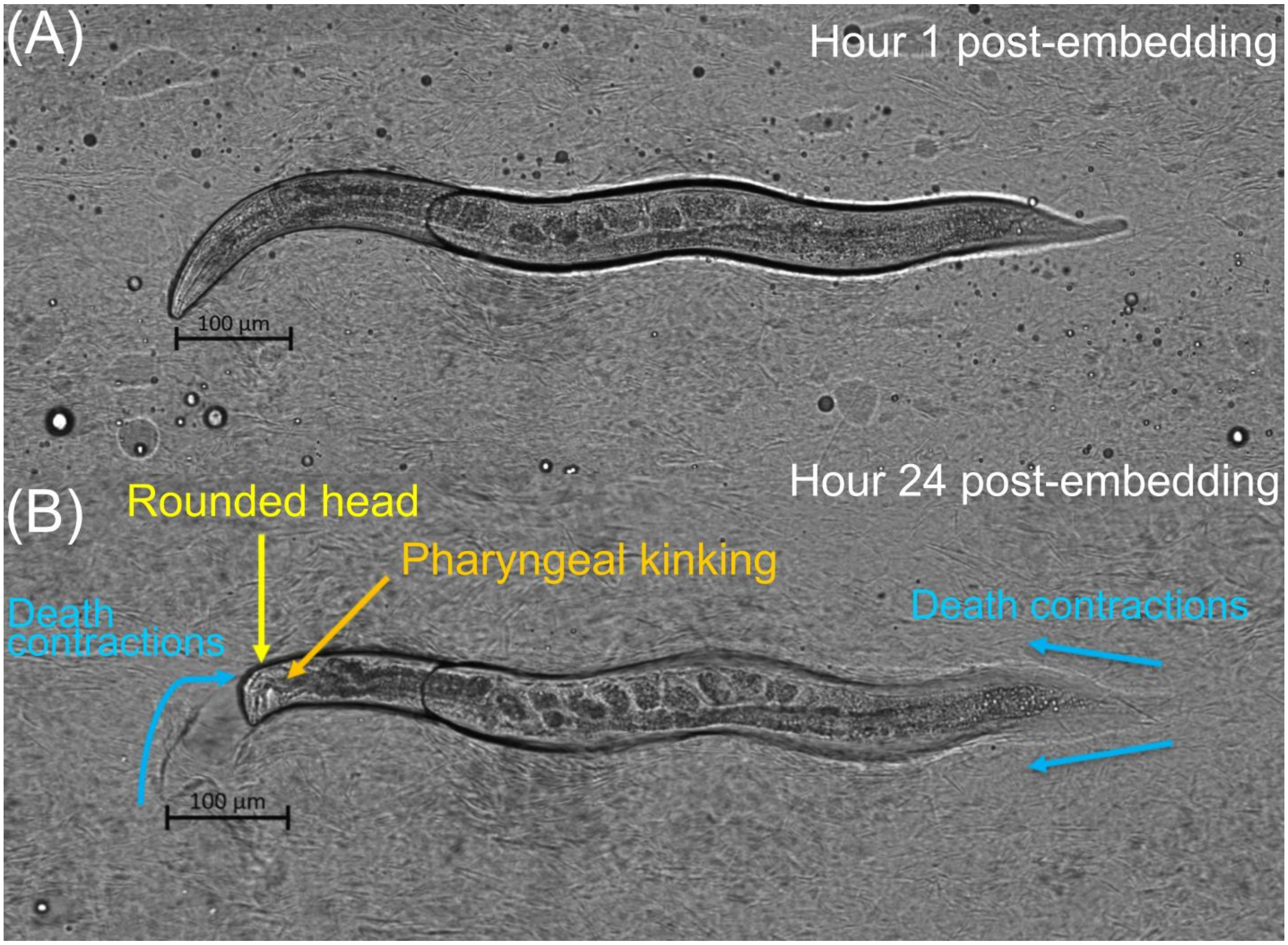

Figure S6: Investigating sample integrity and morphology changes of *C. elegans* embedded in petrolatum. (A) Initial embedding imaging of gravid day 1 adult *C. elegans* at the 1-hour post-embedding time point reveals no disturbed morphology of the worm. (B) Death contractions and pharyngeal kinking indicative of intestinal necrosis observed at the 24-hour post-embedding time point. Commonly observed morphological changes are indicated in blue, yellow and orange. Black scale bars indicate 100 µm.

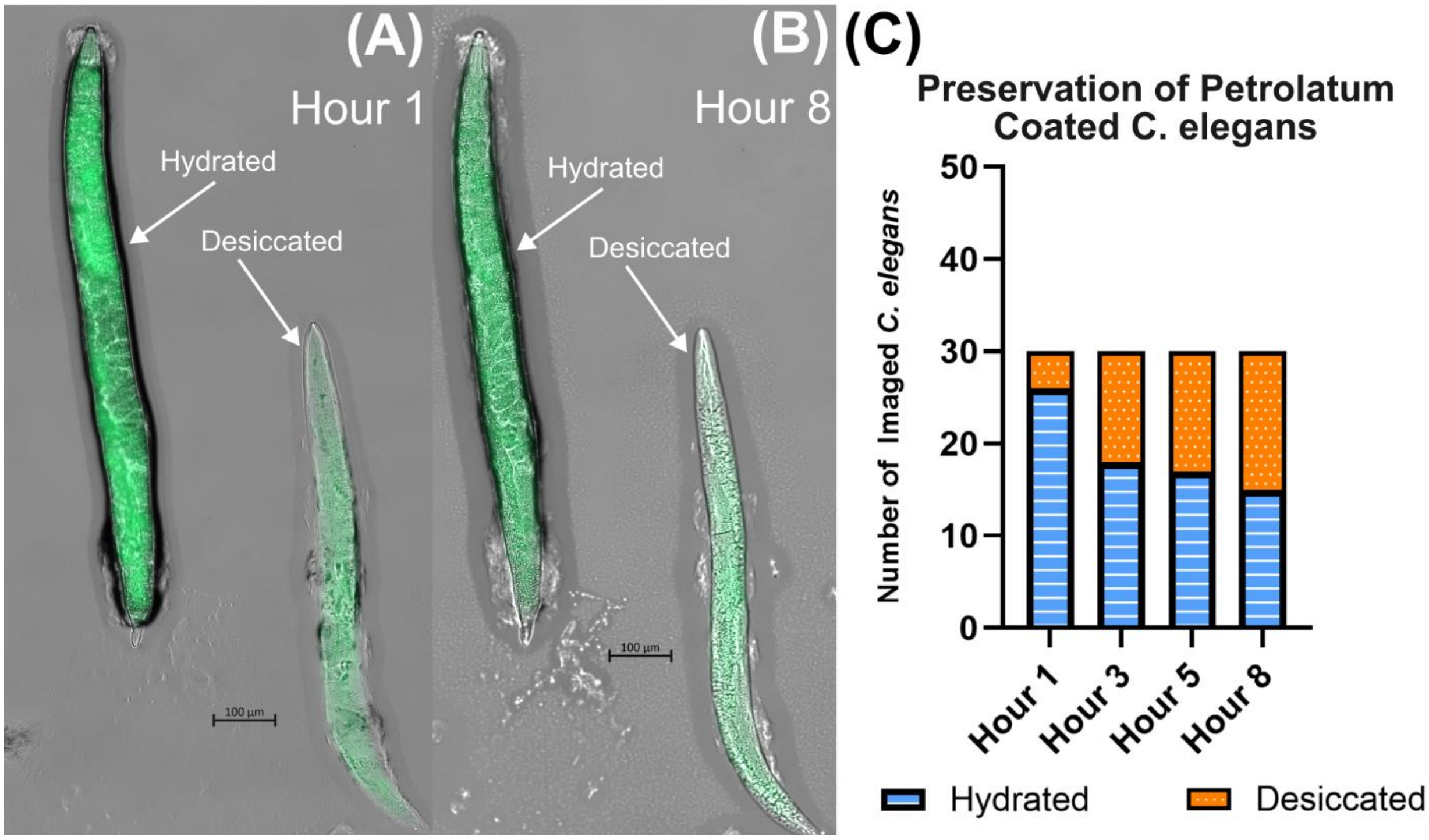

Figure S7: Time-series evaluation of preservation capabilities of petrolatum-coated *C. elegans*. (A) Microscopy imaging of two gravid day 1 adult SJ4144 *C. elegans* at the 1-hour post-coating time point, (B) and at the 8-hour post-coating time point. Hydrated and desiccated worms are indicated by white arrows. Immediate desiccation of coated worms is due to unintentional removal of petrolatum coating during slide transfer. (C) *C. elegans* preservation efficacy over an 8-hour period at ambient conditions. Orange bars with white spotted pattern represent the number of desiccated worms and blue bars with white horizontal stripes represent the number of hydrated worms imaged. A total of n=30 worms were imaged and pooled from 3 different experiment trials. Black scale bars indicate 100 µm.

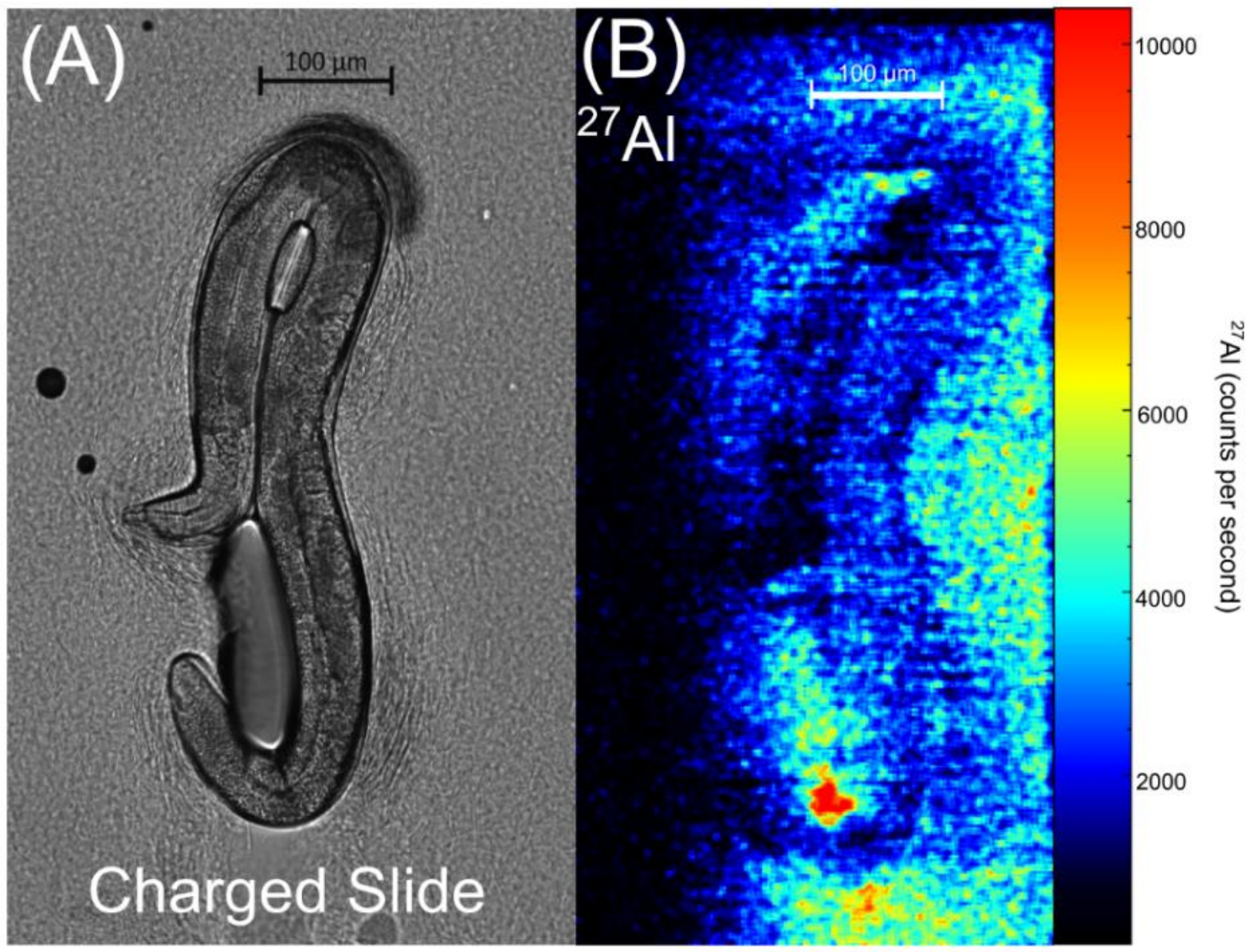

Figure S8: Determining optimal microscope slide for LA-ICP-TOF-MS imaging of *C. elegans* and establishing elemental signals indicative of complete ablation of *C. elegans*. (A) Microscopy and LA-ICP-TOF imaging of a day 1 gravid adult wildtype *C. elegans* embedded in petrolatum. (B) Elemental imaging showing ^{27}Al present in Epredia charged microscope slide glass confirming complete sample ablation. Black scale bars for microscopy images and white scale bars in ion images indicate 100 µm.

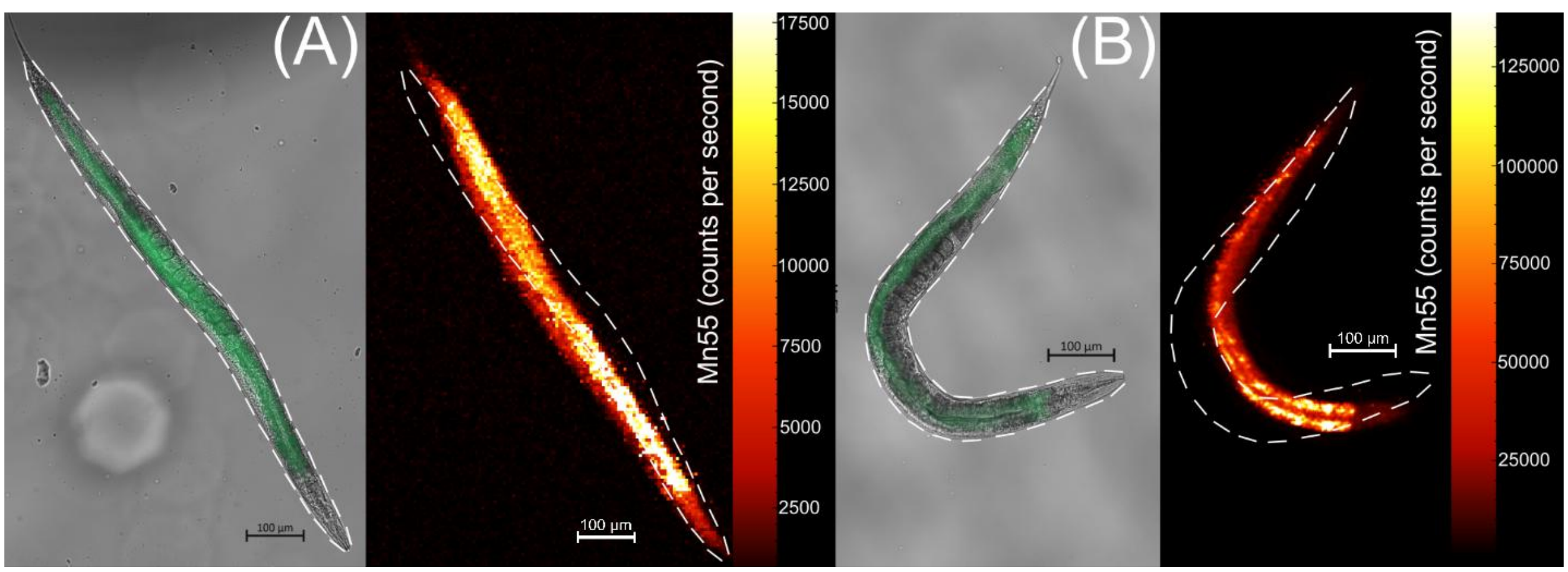

Figure S9: Comparison of laser spot sizes and spatial resolution quality from imaged *C. elegans*. (A) LA-ICP-TOF-MS imaging of day 1 gravid adult SJ4144 *C. elegans* using a 5-µm spot size laser profile, (B) and an effective 2-µm spot size achieved by over-sampling a 5-µm spot size laser profile showing ^{55}Mn distribution. The effective 2-µm spot size achieves a spatial resolution capable of visualizing the intestinal lumen and differentiating intestinal cells. Outlines of imaged *C. elegans* are denoted by the white dashed lines which show that *C. elegans* embedded in OCT change shape and experience morphological changes, possibly due to dessication, from freshly prepared to LA-ICP-TOF-MS imaging. Black scale bars for microscopy images and white scale bars in ion images indicate 100 µm.

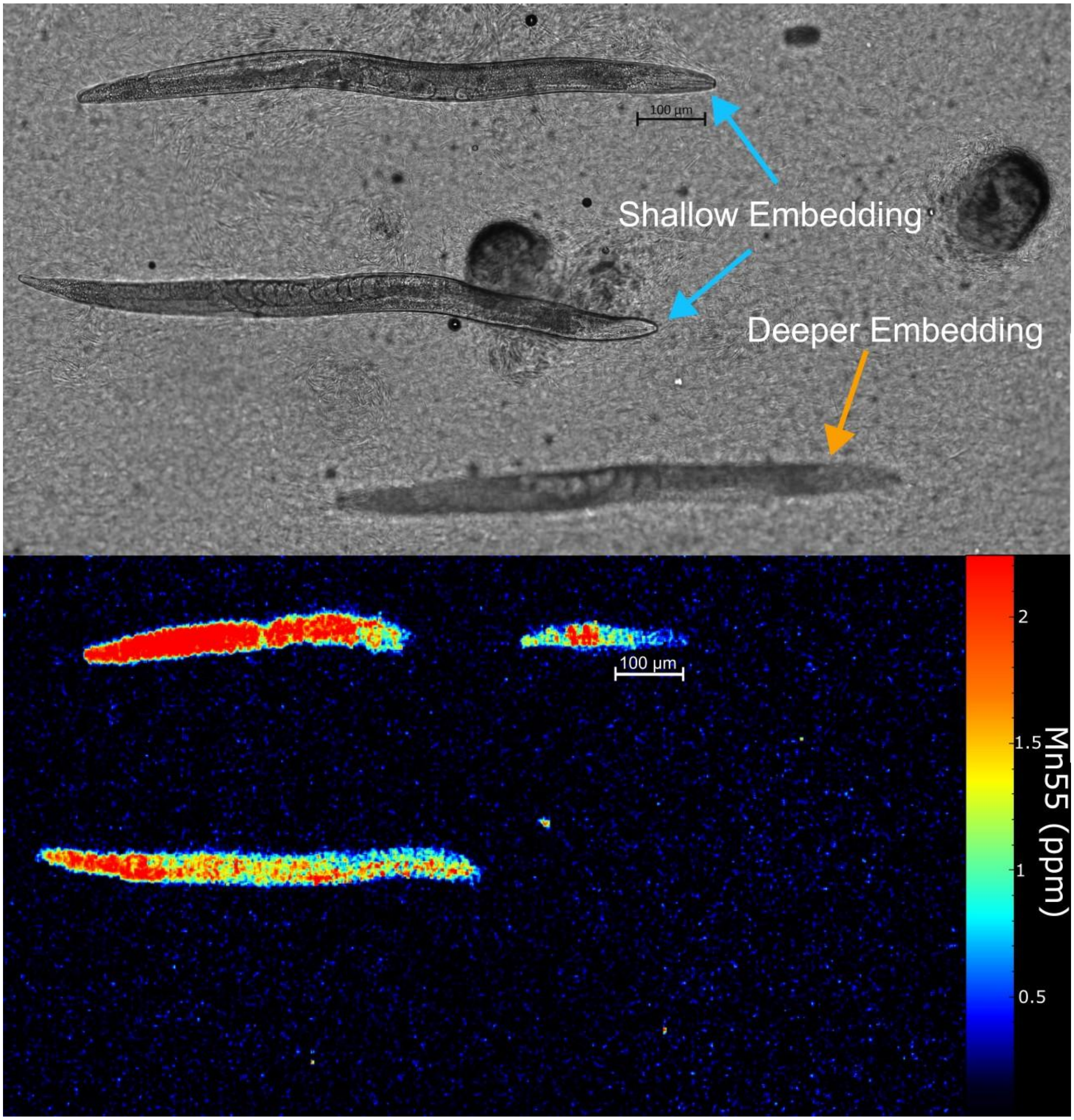

Figure S10: Vaseline shows laser ablation inconsistencies during LA-ICP-TOF-MS imaging. Producing in a ~100 µm embedding layer induces too much variation in *C. elegans'* vertical positioning, requiring further layering height optimization to improve imaging consistency of petrolatum-embedded worms. Black scale bars for microscopy images and white scale bars in ion images indicate 100 µm.

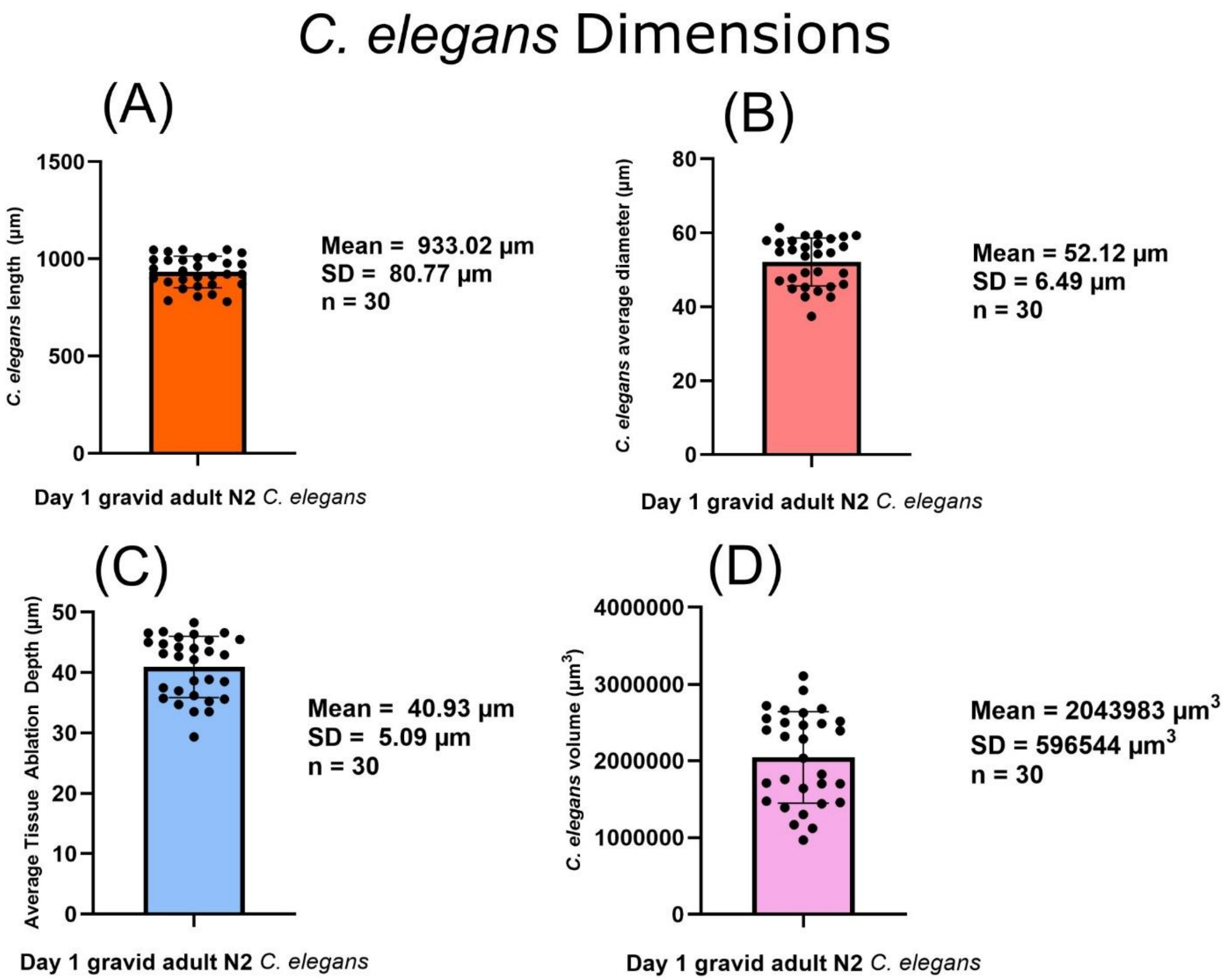

Figure S11: Dimensional analysis of *C. elegans* using the InteredgeDistance macro for ImageJ. (A) Day 1 adult N2 *C. elegans* were sampled within 4 hours of entering adulthood with their length, (B) average diameter across the whole body, (C) average tissue ablation depth, (D) and volumes measured by ImageJ. Average tissue ablation depth was used as reference for determining the gelatin standard sectioning thickness for quantitative LA-ICP-TOF-MS imaging. Dimensions were acquired from *n* = 30 *C. elegans* embedded in petrolatum.

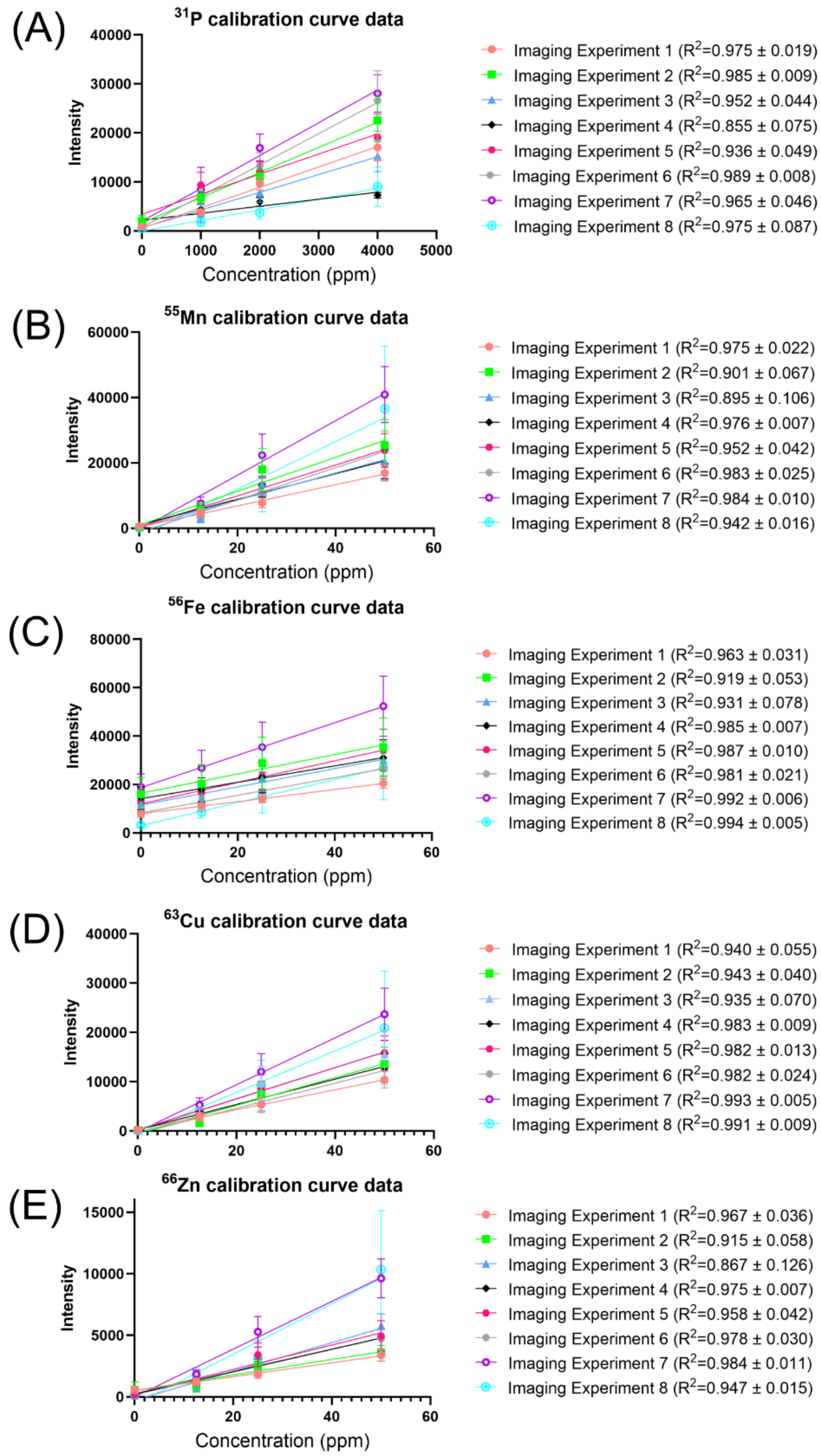

Figure S12: Raw calibration curve data for all quantitative LA-ICP-TOF-MS imaging of *C. elegans.* (A) ^{31}P , (B) ^{55}Mn, (C) ^{56}Fe, (D) ^{63}Cu, (E) and ^{66}Zn calibration curves visualizing standard curves from multi-day imaging experiments. R^2 averages and standard deviations were calculated from the R^2 values of repeated gelatin scan measurements throughout individual runs. All imaging runs were performed on different days to assess inter-day variance. Data not background subtracted and forced through origin (0,0) to visualize blank intensity drift for all measured elements.

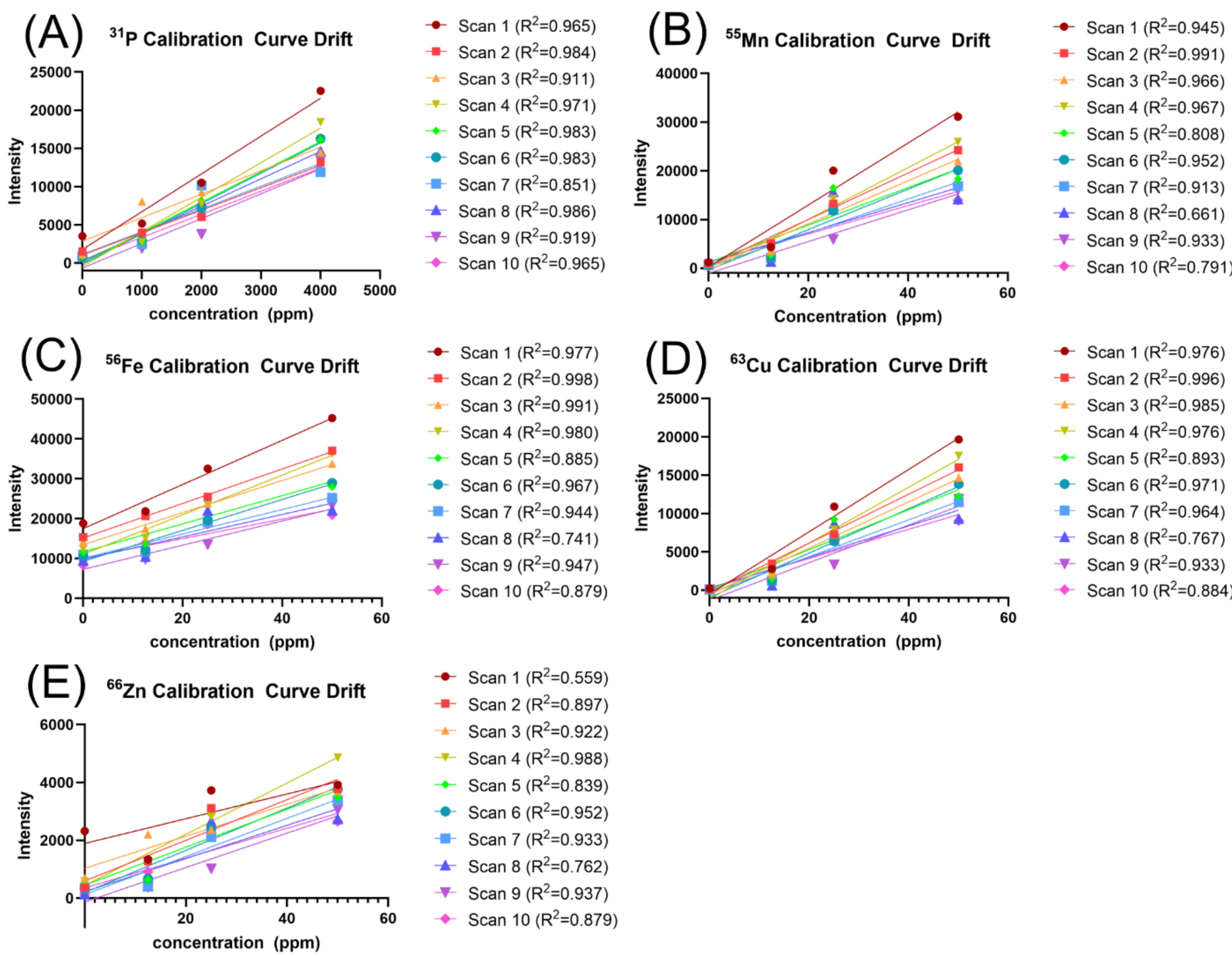

Figure S13: Calibration standard scan lines during a single LA-ICP-TOF-MS imaging experiment showing calibration sensitivity drift for all elements. (A) ^{31}P, (B) ^{55}Mn, (C) ^{56}Fe, (D) ^{63}Cu, (E) and ^{66}Zn show both intensity and sensitivity drift throughout an imaging run. Instrument sensitivity drift contributes to inflated relative standard deviations of pooled calibration standard replicates. Data not background subtracted and forced through origin (0,0) to visualize blank intensity drift for all measured elements.

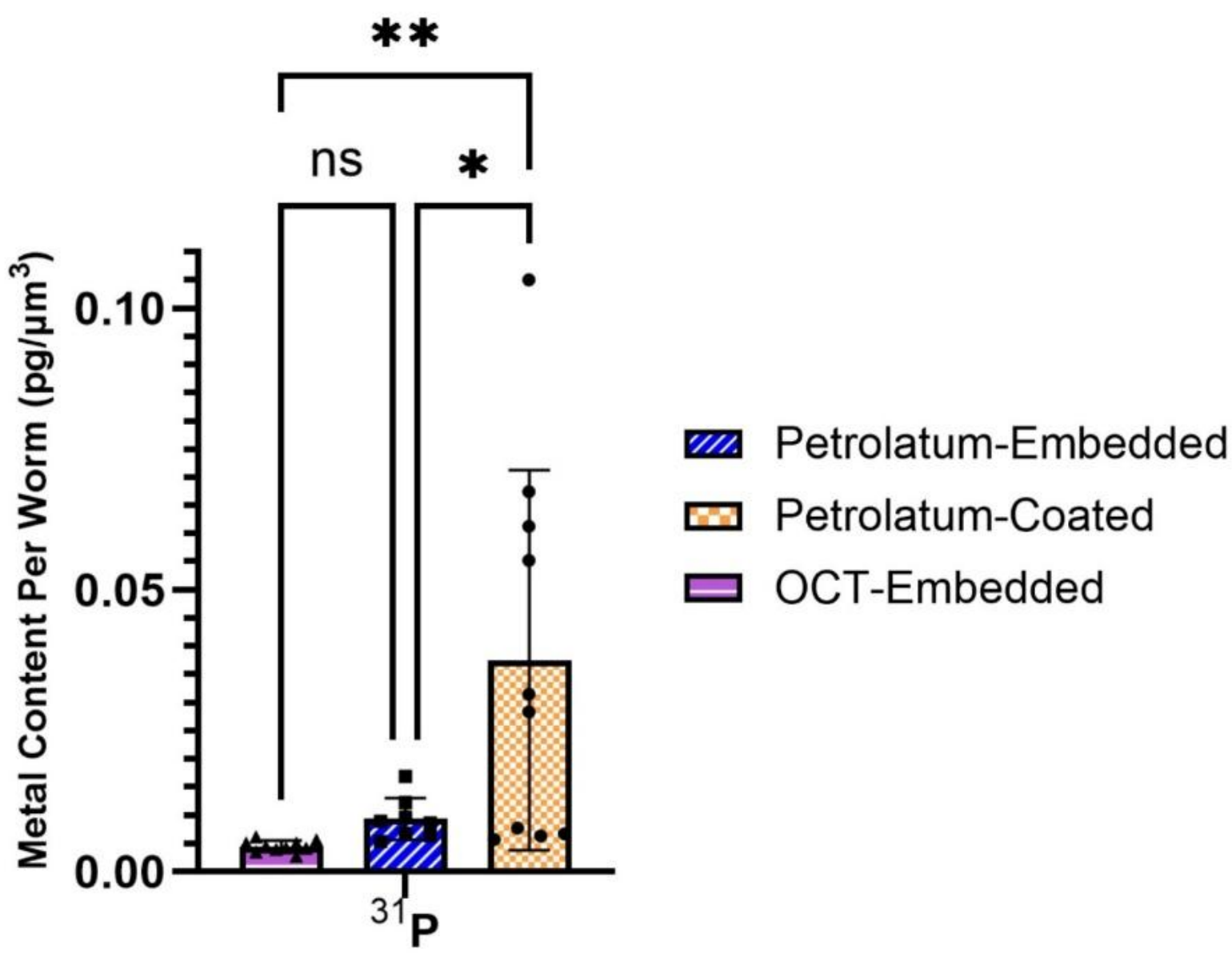

Figure S14: ^{31}P content normalized to individual *C. elegans* volume. ^{31}P content was measured to evaluate if worms were sufficiently rinsed of phosphate content present in M9 buffer during collection. OCT-embedded and petrolatum-embedded groups were found to be sufficiently cleaned of residual M9 buffer, yielding statistically similar content. 6 worms from the petrolatum-coated showed high levels of ^{31}P indicative of residual phosphate-containing M9 buffer on the surface of their bodies. *= $p<0.05$, **=$p<0.01$, One-way ANOVA; Tukey's HSD.

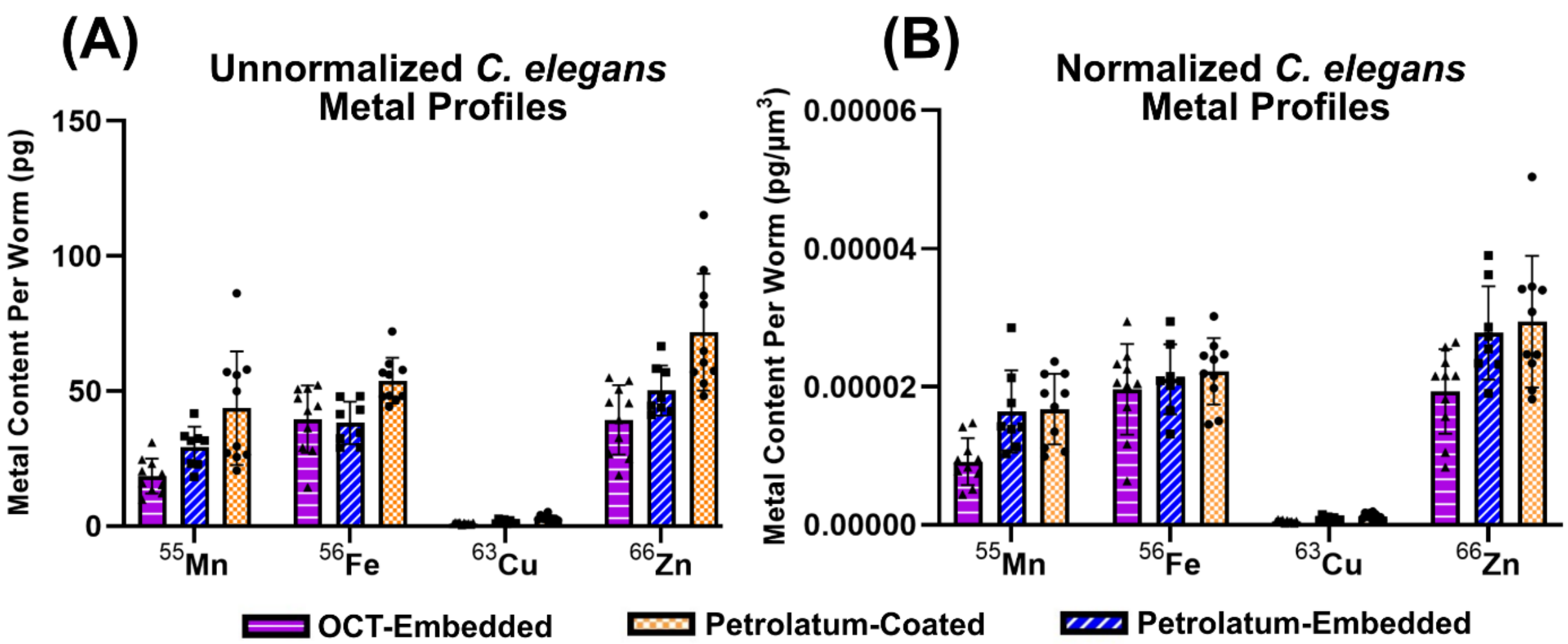

Figure S15: Total metal content determination in *C. elegans* by quantitative LA-ICP-TOF-MS imaging prepared using different sample preparation techniques. (A) Unnormalized metal content from *C. elegans* reported as picograms (pg) show large differences between all three sample preparation techniques. (B) Metal content normalized to the imaged worm's calculated volume (pg/µm^3) reduces metal content differences due to developmental variation.

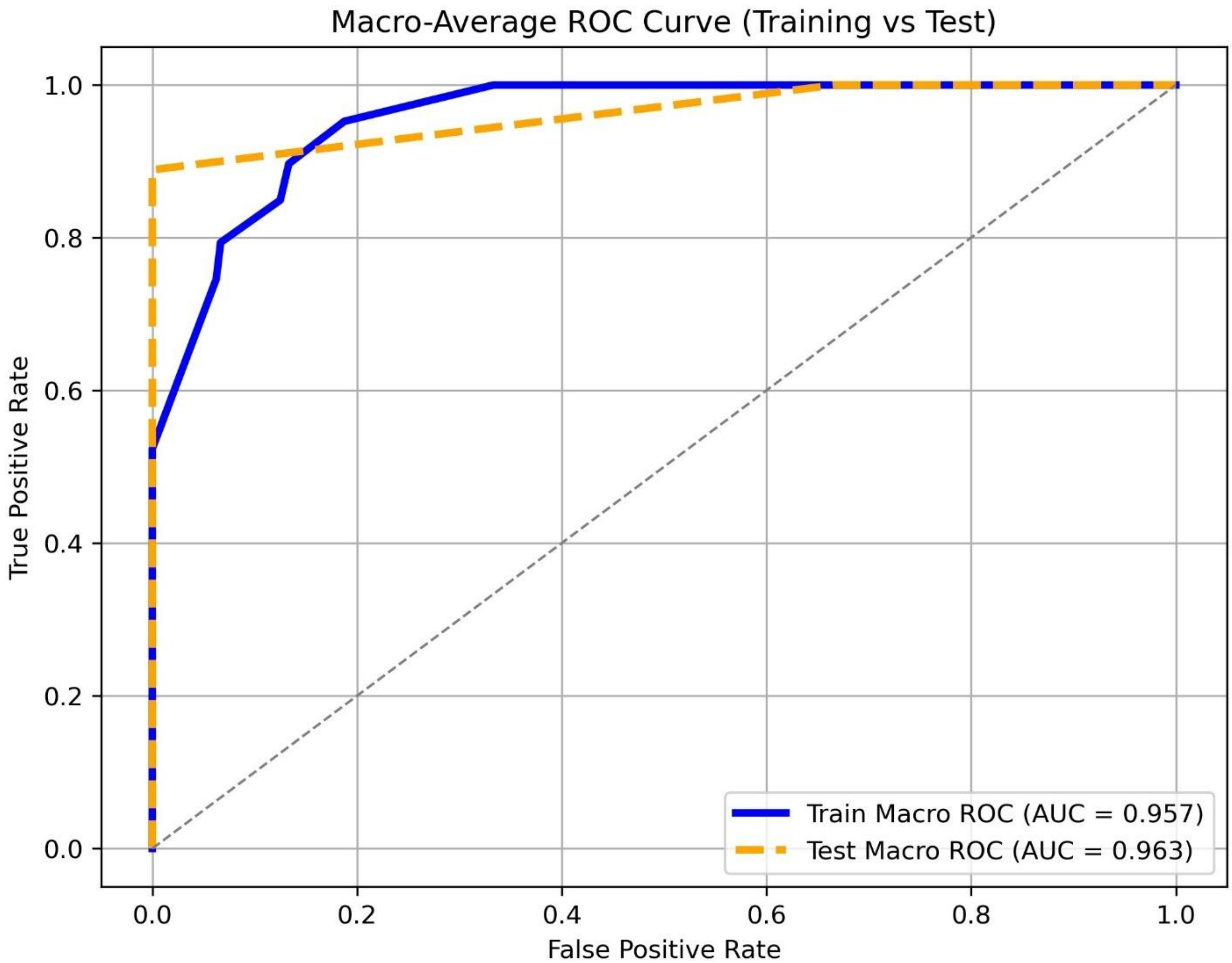

Figure S16: Evaluation of LDA model performance for class separation of OCT-embedded, petrolatum-embedded, and petrolatum-coated *C. elegans* used in post-hoc testing of MANOVA data. Both training and test sets yielded AUC values above 0.95 suggesting the model accurately separated classes. Training split used 80% of data (22 randomly selected worms) and test split used 20% of data (6 randomly selected worms).
